# A sense-antisense RNA interaction promotes breast cancer metastasis via regulation of NQO1 expression

**DOI:** 10.1038/s43018-023-00554-7

**Published:** 2023-05-11

**Authors:** Bruce Culbertson, Kristle Garcia, Daniel Markett, Hosseinali Asgharian, Li Chen, Lisa Fish, Albertas Navickas, Johnny Yu, Brian Woo, Arjun Scott Nanda, Benedict Choi, Shaopu Zhou, Joshua Rabinowitz, Hani Goodarzi

**Affiliations:** 1 Department of Biochemistry and Biophysics, University of California, San Francisco, San Francisco, CA USA; 2 Department of Urology, University of California, San Francisco, San Francisco, CA USA; 3 Helen Diller Family Comprehensive Cancer Center, University of California, San Francisco, San Francisco, CA USA; 4 Bakar Computational Health Sciences Institute, University of California, San Francisco, San Francisco, CA USA; 5 Shanghai Key Laboratory of Metabolic Remodeling and Health, Institute of Metabolism & Integrative Biology, Fudan University, Shanghai, China; 6 Department of Chemistry, Lewis Sigler Institute for Integrative Genomics, Princeton, NJ USA; 7 Ludwig Institute for Cancer Research, Princeton, NJ USA

**Keywords:** Breast cancer, Mechanisms of disease, Gene expression, Cancer

## Abstract

Antisense RNAs are ubiquitous in human cells, yet their role is largely unexplored. Here we profiled antisense RNAs in the MDA-MB-231 breast cancer cell line and its highly lung metastatic derivative. We identified one antisense RNA that drives cancer progression by upregulating the redox enzyme NADPH quinone dehydrogenase 1 (NQO1), and named it NQO1-AS. Knockdown of either NQO1 or NQO1-AS reduced lung colonization in a mouse model, and investigation into the role of NQO1 indicated that it is broadly protective against oxidative damage and ferroptosis. Breast cancer cells in the lung are dependent on this pathway, and this dependence can be exploited therapeutically by inducing ferroptosis while inhibiting NQO1. Together, our findings establish a role for NQO1-AS in the progression of breast cancer by regulating its sense mRNA post-transcriptionally. Because breast cancer predominantly affects females, the disease models used in this study are of female origin and the results are primarily applicable to females.

## Abstract (web summary)

Culbertson et al. profile antisense RNAs using a newly developed computational pipeline and identify NQO1-AS as contributor to breast cancer progression and lung metastasis through regulation of the redox enzyme NQO1.

## Main

Metastasis is a disease of disordered gene expression^1,2^. During cancer progression, changes in gene expression patterns have a profound impact on nearly every aspect of the cell, promoting uninhibited spread. For this reason, understanding the cellular pathways that underlie gene expression changes is a necessary step toward developing therapies that target metastasis. Cancer cells often co-opt post-transcriptional regulatory mechanisms to achieve pathological expression of cellular pathways that impact metastasis^3–5^. While RNA-binding proteins^6,7^ and microRNAs (miRNAs)^8^ have been the focus of many studies, interactions between RNA transcripts may also have a regulatory role^9–11^. However, the extent to which RNA–RNA interactions influence gene expression and regulate cell physiology and human disease are unknown.

Antisense RNAs have great regulatory potential because they readily form duplexes with RNAs transcribed from their complementary sense strands. They are also ubiquitous in human cells; it is estimated that approximately 30% of human protein-coding genes have a corresponding antisense RNA^12^. Yet, little is known about their regulatory functions in the cell. Members of this family impact gene expression through different mechanisms, including DNA methylation^13^, chromatin modification^14^ and RNA degradation^15^. Some antisense RNAs have also been associated with tumorigenesis and a select few have a functional role in cancer progression^16^. Still, the extent to which antisense RNAs contribute to the regulation of gene expression in cancer is poorly understood.

Given their ubiquity and potential regulatory role, an unbiased study of antisense RNA in cancer is needed. To this end, we developed a pipeline to profile antisense RNAs and applied it to an established model of breast cancer metastasis. Based on analyses of this dataset, we identified an antisense RNA whose upregulation promotes breast cancer metastasis. This RNA is complementary to the 3′-UTR of NADPH quinone dehydrogenase 1 (*NQO1*); therefore, we named it NQO1-AS. By binding directly to its complementary region, NQO1-AS stabilizes the *NQO1* mRNA, upregulating the NQO1 gene product, an enzyme that protects cells against oxidative stress. Therefore, NQO1-AS enables breast cancer cells to become resistant to oxidative damage, leading to a decrease in sensitivity to ferroptosis. During metastasis to the lung, cells are dependent on this NQO1 pathway; we show that downregulation of either NQO1 or NQO1-AS significantly decreases lung metastatic burden in a mouse model. Furthermore, we found that adding a ferroptosis-inducing agent can enhance a therapeutic regimen targeting NQO1.

## Results

### Annotation of antisense RNAs occluded by sense transcripts

**Extended Data Fig. 1:**
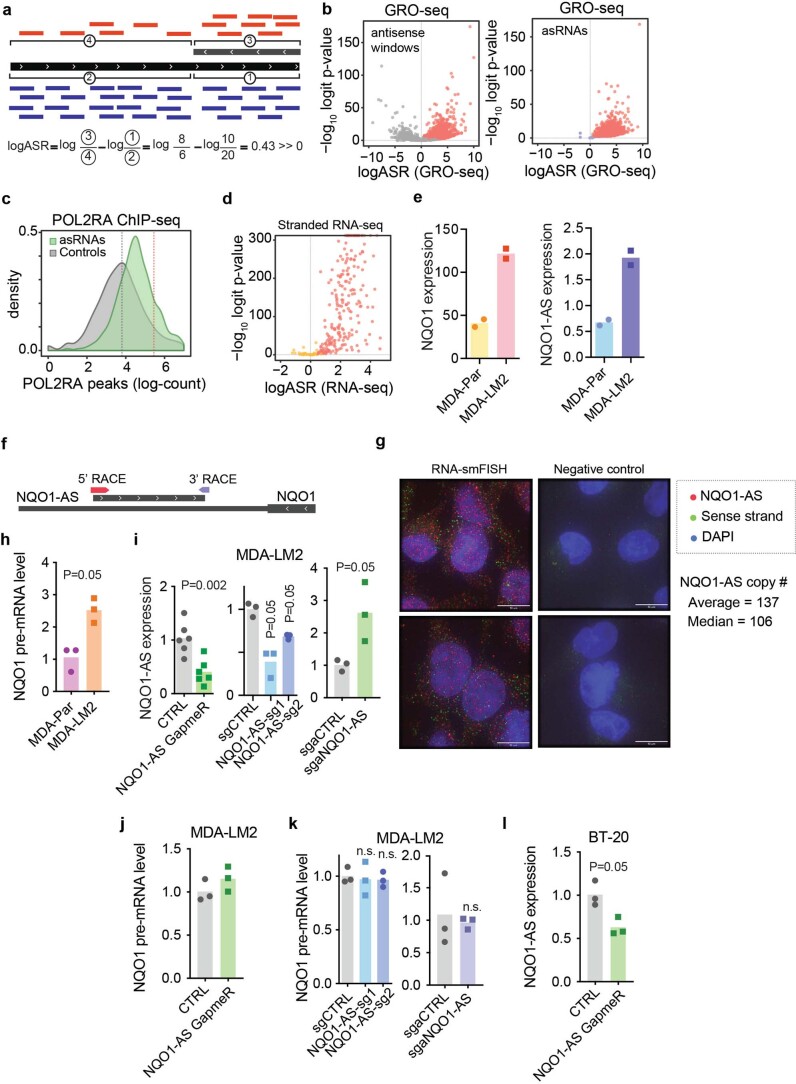
Discovery and annotation of antisense RNAs. (a) Schematic showing sense and antisense read distribution in IRIS and the calculation of logASR. (b) Volcano plots showing windows of high antisense activity (left) and annotated asRNAs (right) based on GRO-seq data. (c) Prevalence of asRNAs and negative controls in POL2RA ChIP-seq data from ENCODE. Red line represents threshold above which there is strong evidence for RNA Pol II binding across multiple cell lines. (d) Volcano plot showing logASR distribution from stranded RNA-seq in MDA-Par and MDA-LM2 cells. (e) Relative NQO1 (left) and NQO1-AS (right) expression in MDA-Par and MDA-LM2 cells measured by RNA seq (TPM). N = 2 independent cell cultures. (f) Tracks indicating the 5’ (red) and 3’ (purple) ends of NQO1-AS, as identified by 5’ and 3’ RACE, respectively. (g) Representative images from RNA-smFISH in MDA-LM2 cells, using probes targeted to NQO1-AS (red) and NQO1 (green). (h) Relative NQO1 pre-mRNA level in MDA-Par and MDA-LM2 cells measured by qPCR. N = 3 independent cell cultures. (i) Relative NQO1-AS expression in MDA-LM2 cells with GapmeR-mediated NQO1-AS knockdown (N = 6 independent cell cultures), CRISPRi-mediated NQO1-AS knockdown (N = 3 independent cell cultures), and CRISPRa-mediated NQO1-AS overexpression (N = 3 independent cell cultures), measured by qRT-PCR. (j) Relative NQO1 pre-mRNA level in MDA-LM2 cells with Gapmer-mediated NQO1-AS knockdown, measured by qRT-PCR. N = 3 independent cell cultures. (k) Relative NQO1 pre-mRNA level in MDA-LM2 cells with CRISPRi-mediated NQO1-AS knockdown and CRISPRa-mediated NQO1-AS overexpression, measured by qRT-PCR. N = 3 independent cell cultures. (l) Relative NQO1-AS expression in BT-20 cells with GapmeR-mediated NQO1-AS knockdown, measured by qRT-PCR. N = 3 independent cell cultures. All *P* values were calculated using one tailed Mann-Whitney U tests. Source data

We recently developed identification of RNA antisense species (IRIS), a computational pipeline that integrates data from RNA sequencing (RNA-seq), global run-on sequencing (GRO-seq) and RNA polymerase II (RNAP II) chromatin immunoprecipitation followed by sequencing (ChIP–seq) to identify and quantify antisense RNAs. We applied this pipeline to a well-characterized model of breast cancer progression, the MDA-MB-231 parental cell line (MDA-Par) and its highly lung metastatic derivative MDA-LM2 (refs. ^1,3,17^). We first performed GRO-seq in MDA-Par cells to capture the footprint of transcriptionally active RNAP II across the transcriptome. We used the resulting stranded data to ask whether there are actively transcribed loci that show higher coverage of the antisense strand than expected by chance. We used a sliding window of 500 nt and a statistical framework built on logistic regression to calculate a *P* value for antisense transcription as measured by the number log ratio of antisense to sense reads in each window relative to the background, a quantity we named logASR (Extended Data Fig. 1a). We used the sequences with significantly positive logASR values (logASR > 0.5; false discovery rate (FDR)-adjusted *P* < 0.01) to annotate parts of the transcriptome that show significant antisense RNA signal (Extended Data Fig. 1b); we generated a set of negative annotations as a control group (logASR < 0; FDR-adjusted *P* > 0.5) for the subsequent steps. To determine the level of RNAP II binding in our regions of interest, we took advantage of POLR2A ChIP–seq datasets from ENCODE, using our negative control set to identify a threshold above which there is strong evidence of RNAP II activity across many cell lines (Extended Data Fig. 1c). As shown in Extended Data Fig. 1d, we identified approximately 300 loci with strong evidence for antisense RNA transcription. Finally, we performed stranded RNA-seq on MDA-Par and MDA-LM2 cells and used the resulting dataset to calculate the logASR values for these 300 regions (Extended Data Fig. 1d). We selected a logASR of 1.0 and adjusted *P* value of 1 × 10^−5^ for our final annotation of 262 antisense RNAs expressed in the MDA-MB-231 background. Of these, 20 overlapped previously annotated antisense RNAs or pseudogenes and the rest were not previously reported.

**Fig. 1:**
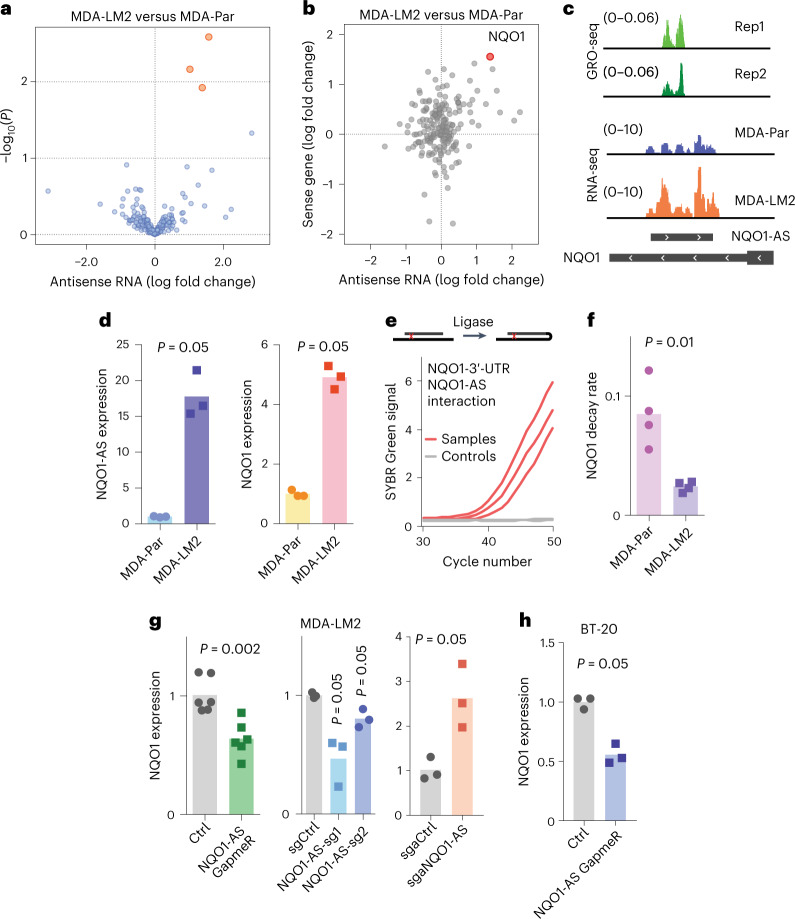
NQO1-AS interacts with the 3′-UTR of *NQO1* and regulates *NQO1* expression. **a**, Volcano plot showing antisense RNAs in MDA-Par and MDA-LM2 cells. **b**, Fold change in expression level of antisense RNAs and their corresponding sense RNAs in MDA-LM2 cells relative to MDA-Par cells. *NQO1* is indicated by the red dot. **c**, Tracks showing GRO-seq peaks in MDA-LM2 cells, along with RNA-seq in the MDA-LM2 and MDA-Par cell lines. **d**, RT–qPCR of NQO1-AS and *NQO1* in MDA-Par and MDA-LM2 cell lines. *n* = 3 independent cell cultures. **e**, Psoralen-mediated RNA crosslinking followed by ligation. RT–qPCR was used to detect the interactions between the *NQO1* sense 3′-UTR with NQO1-AS RNA. Samples without the proximity ligation step were used as controls. **f**, Relative *NQO1* decay rate in MDA-Par and MDA-LM2 cell lines from our metabolomic pulse-chase labeling of these cell lines^3^. *n* = 4 independently treated cell cultures. **g**, Relative *NQO1* mRNA levels in MDA-LM2 cells with GapmeR-mediated NQO1-AS knockdown (*n* = 6 independent cell cultures), CRISPRi-mediated NQO1-AS suppression (*n* = 3 independent cell cultures) and CRISPRa-mediated NQO1-AS overexpression (*n* = 3 independent cell cultures), measured using RT–qPCR. sg, single-guide. **h**, Relative *NQO1* mRNA levels in BT-20 cells after GapmeR-mediated NQO1-AS knockdown, measured using RT–qPCR. *n* = 3 independent cell cultures. All *P* values were calculated using a one-tailed Mann–Whitney *U*-test. Source data

We then asked whether any of these antisense RNAs are associated with the increased metastatic capacity of MDA-LM2 cells. Using our RNA-seq data from MDA-Par and MDA-LM2 cells, we performed differential gene expression analysis focused on our annotated antisense RNA species. As shown in Fig. 1a, our analysis revealed three antisense RNAs that were significantly upregulated in the highly metastatic line. Among these, we chose to focus on one uncharacterized RNA that is transcribed from the antisense strand of the gene *NQO1*. This antisense RNA, which is annotated as CTD-2033A16.1, overlaps the 3′-UTR of *NQO1*, which we named NQO1-AS. We believed NQO1-AS to be a promising candidate for follow-up for several reasons. First, both the antisense RNA and its sense mRNA (*NQO1*) were simultaneously upregulated in the highly metastatic MDA-LM2 cells (Fig. 1b–d and Extended Data Fig. 1e). Second, they are transcribed from distinct promoters about 20 kb apart, suggesting that their correlated expression is not simply due to a shared promoter. Third, the location of the NQO1-AS complementary sequence within the 3′-UTR of *NQO1* raised the possibility of a regulatory interaction between the two RNA species. Taken together, these features prompted us to ask if NQO1-AS has a functional role in breast cancer progression by driving the post-transcriptional upregulation of *NQO1*.

### NQO1-AS binds and stabilizes the NQO1 sense transcript

First, we performed 5′ and 3′ rapid amplification of complementary DNA (cDNA) ends (RACE) and confirmed that the observed 5′ and 3′ ends of the molecule matched our annotation from IRIS (Extended Data Fig. 1f). We then performed single-molecule RNA fluorescence in situ hybridization (smRNA-FISH) using probes targeting either NQO1-AS or the sense *NQO1* mRNA (Extended Data Fig. 1g). To assess the functional relationship between NQO1-AS and *NQO1*, we asked if we could detect a direct interaction between the two RNA species. For this, we used psoralen crosslinking followed by nuclease digestion and proximity RNA ligation^18^. In this assay, the presence of ligase-induced artificial junctions revealed in vivo duplex formation between the *NQO1* sense and antisense RNAs (Fig. 1e). We next asked whether the upregulation of *NQO1* in MDA-LM2 cells was due to increased transcription or mRNA stabilization. Whole-genome RNA stability measurements in MDA-Par and MDA-LM2 cells^3^ revealed that *NQO1* mRNA is significantly stabilized (approximately fourfold) in MDA-LM2 cells relative to their poorly metastatic parental line (Fig. 1f). The *NQO1* transcription rate, as measured by pre-mRNA levels, was elevated approximately twofold in MDA-LM2 cells (Extended Data Fig. 1h), but this elevation was insufficient to account for the degree of NQO1 upregulation. These results indicate that the increase in *NQO1* mRNA in the highly metastatic cell line is due in part to post-transcriptional stabilization. To test whether NQO1-AS binding stabilizes *NQO1*, we used locked nucleic acid (LNA) GapmeRs for targeted knockdown of NQO1-AS (Extended Data Fig. 1i). We observed that on GapmeR-induced knockdown of NQO1-AS, *NQO1* mRNA levels decreased by a similar magnitude (Fig. 1g), whereas *NQO1* pre-mRNA levels were unchanged (Extended Data Fig. 1j). To verify this result, we repeated this experiment using CRISPR interference (CRISPRi) to knock down NQO1-AS (Extended Data Fig. 1i). Once again, *NQO1* pre-mRNA levels were unaffected (Extended Data Fig. 1k) and we saw a decrease in mature *NQO1* mRNA (Fig. 1g). To confirm that this result was not specific to the MDA-LM2 background, we used LNA GapmeRs to knock down NQO1-AS in the BT-20 cell line (Extended Data Fig. 1l), a triple-negative breast cancer line that expresses NQO1-AS at a high level. Again, we observed a corresponding decrease in *NQO1* expression (Fig. 1h). Next, we asked if overexpression of NQO1-AS was sufficient to upregulate NQO1. We performed this experiment in MDA-LM2 cells using CRISPR activation (CRISPRa) (Extended Data Fig. 1i); consistent with our previous results, we saw a corresponding increase in mature *NQO1* mRNA (Fig. 1g) with no change in pre-mRNA levels (Extended Data Fig. 1k).

### NQO1-AS binding modulates poly(A) site selection

**Fig. 2:**
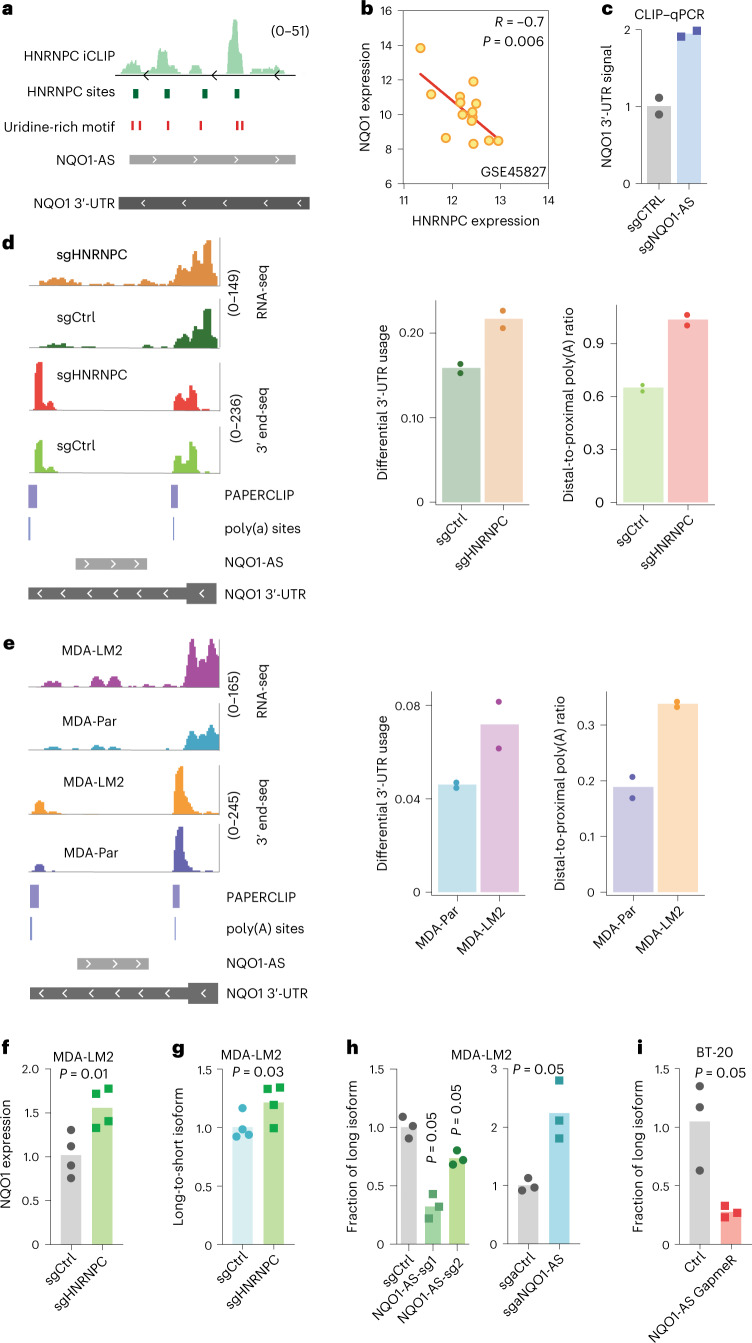
NQO1-AS masking of HNRNPC binding sites modulates poly(A) site selection. **a**, Tracks showing the relationship between HNRNPC iCLIP data, known HNRNPC binding sites and uridine-rich motifs along the *NQO1* 3′-UTR. **b**, Pearson correlation between NQO1 and HNRNPC expression in multiple independent datasets. **c**, CLIP–qPCR with immunoprecipitation of HNRNPC in NQO1-AS knockdown and control MDA-LM2 cells. qPCR primers are targeting the *NQO1* 3′-UTR. *n* = 2 independent cell cultures. **d**, Read distribution of RNA-seq and 3′ end-seq in MDA-Par cells with and without knockdown of HNRNPC, along with bar graph representation of differential 3′-UTR usage and distal-to-proximal poly(A) ratio. *n* = 2 independent cell cultures. **e**, Read distribution of RNA-seq and 3′ end-seq in MDA-Par and MDA-LM2 cells, along with bar graph representation of differential 3′-UTR usage and distal-to-proximal poly(A) ratio. *n* = 2 independent cell cultures. **f**, Relative NQO1 expression in HNRNPC knockdown and control MDA-LM2 cells, measured by RT–qPCR. *n* = 4 independent cell cultures. **g**, Relative *NQO1* long-to-short isoform ratios in HNRNPC knockdown and control MDA-LM2 cells, measured using RT–qPCR. *n* = 4 independent cell cultures. **h**, Relative *NQO1* long-to-short isoform ratios in NQO1-AS knockdown or overexpression MDA-LM2 and control cells, measured using RT–qPCR. *n* = 3 independent cell cultures. **i**, Relative *NQO1* long-to-short isoform ratios in BT-20 cells with GapmeR-mediated NQO1-AS knockdown, measured using RT–qPCR. *n* = 3 independent cell cultures. All *P* values were calculated using a one-tailed Mann–Whitney *U*-test. Source data

**Extended Data Fig. 2:**
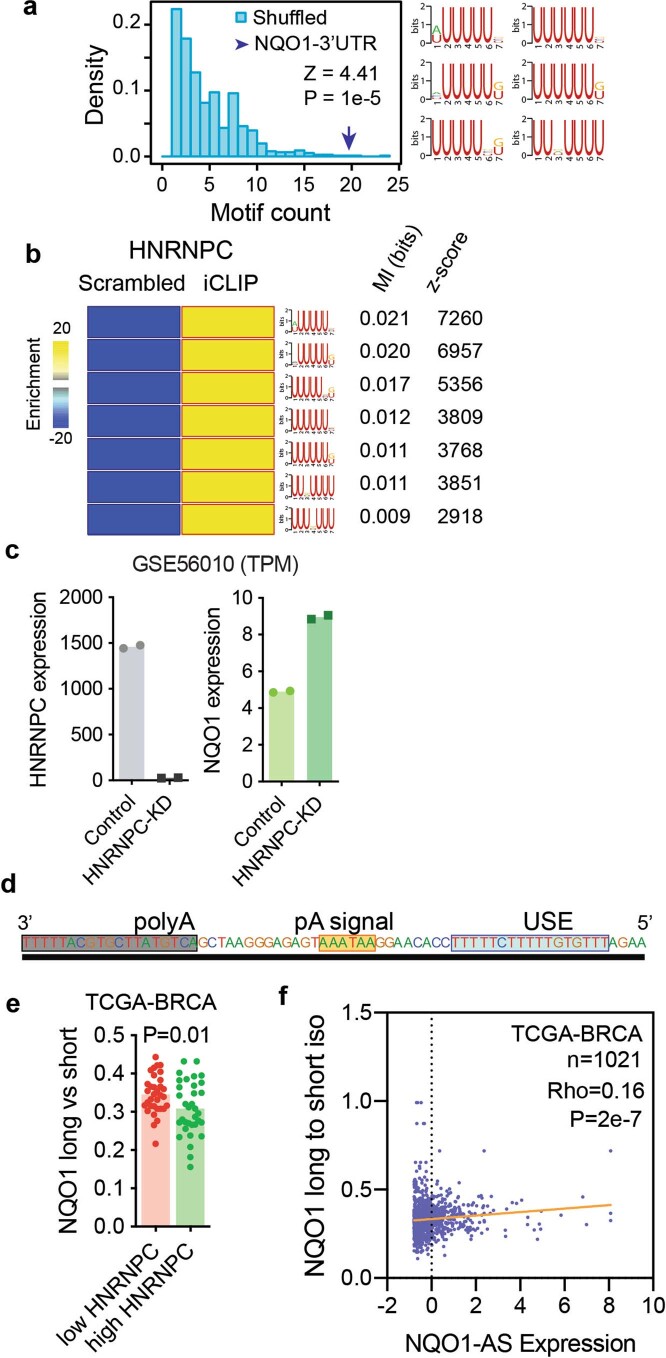
NQO1-AS binding masks HNRNPC binding sites which modulates polyA site selection. (a) U-rich motif density in *NQO1* 3’UTR region complementary to NQO1-AS (purple arrow) relative to scrambled sequences with the same dinucleotide frequency (blue). (b) Heat map showing U-rich motif enrichment in HNRNPC iCLIP data relative to scrambled sequences. (c) Relative HNRNPC (left) and NQO1 (right) expression in HNRNPC knockdown and control (GSE56010; HEK293T) cells measured by RNA-seq. N = 2 independent cell cultures. (d) Region of *NQO1* 3’UTR highlighting two canonical polyadenylation sites. (e) Relative long vs short *NQO1* isoform ratio in cells with high or low HNRNPC expression from TCGA-BRCA dataset (n = 33 and 24 patients respectively; ~top and bottom 5% of HNRNPC expression values). (f) Spearman correlation between *NQO1* long to short isoform ratio and NQO1-AS expression in TCGA-BRCA dataset. N = 1021 patients. The *P* value in (a) was calculated using a shuffle test. The *P* value in (e) was calculated using a one-tailed Mann-Whitney U test. Source data

Having established that NQO1-AS stabilizes *NQO1* mRNA by binding to its 3′-UTR, we next sought to understand the mechanism by which this occurs. We hypothesized that NQO1-AS binding masks *cis*-regulatory elements targeted by destabilizing factors. To identify these elements, we searched for motifs overrepresented in the region of the *NQO1* 3′-UTR complementary to NQO1-AS relative to scrambled control sequences. We did not identify any strong recognition sites for known miRNAs; however, we observed a significant enrichment of uridine-rich motifs in this region (Extended Data Fig. 2a). To identify the RNA-binding protein (RBP) that binds these elements, we used publicly available crosslinking and immunoprecipitation (CLIP) followed by the RNA-seq datasets CLIPdb^19^ and ENCODE eCLIP^20^, and our own CLIP datasets^3,4,7,21^. We observed that the uridine-rich motifs in the *NQO1* 3′-UTR were most similar to CLIP-derived heterogenous nuclear ribonuclear protein C (HNRNPC) binding sites (Extended Data Fig. 2b), defined using the HNRNPC individual-nucleotide resolution CLIP (iCLIP) data^22^. Consistent with this analysis, we identified five significant HNRNPC binding sites on the *NQO1* 3′-UTR corresponding to uridine-rich motifs (Fig. 2a). We also observed a negative correlation between HNRNPC and NQO1 levels in multiple independent datasets^23^ (Fig. 2b), suggesting that HNRNPC may act as a post-transcriptional destabilizing factor. Furthermore, we analyzed RNA-seq data from HNRNPC knockdown cells^24^ and observed a significant increase in the expression of NQO1(Extended Data Fig. 2c). To test our hypothesis that NQO1-AS binding masks HNRNPC binding sites, we performed HNRNPC CLIP quantitative PCR (qPCR) in NQO1-AS knockdown and control cells. As expected, we observed significantly higher binding of HNRNPC to the *NQO1* 3′-UTR on NQO1-AS knockdown (Fig. 2c).

Recently, HNRNPC was shown to have a role in regulating transcriptome-wide poly(A) site selection^25–27^. Therefore, we hypothesized that HNRNPC binding might decrease the stability of *NQO1* mRNA by a similar mechanism. Our analyses revealed that the *NQO1* 3′-UTR contains two canonical polyadenylation sites (Extended Data Fig. 2d), which were also present in an experimentally derived poly(A) site dataset^28^. To address whether HNRNPC binding influences poly(A) site selection in *NQO1*, we analyzed The Cancer Genome Atlas (TCGA)-Breast Invasive Carcinoma (BRCA) dataset to determine if there was a difference in the ratio of the long and short *NQO1* isoforms, resulting from proximal or distal poly(A) site selection, in cells with high or low HNRNPC expression. Indeed, we found that the long-to-short isoform ratio was increased in cells with low HNRNPC (Extended Data Fig. 2e). We also found that higher NQO1-AS expression was correlated with a greater long-to-short *NQO1* isoform ratio (Extended Data Fig. 2f). To test these results experimentally, we performed RNA-seq and 3′ end-seq in MDA-Par cells with and without knockdown of HNRNPC^27^ (Fig. 2d). Consistently, we found that HNRNPC knockdown cells had more RNA-seq reads in the portion of the *NQO1* 3′-UTR distal to the proximal poly(A) site than did the controls. Additionally, 3′ end-seq showed significantly higher usage of the distal poly(A) site in the HNRNPC knockdown cells (Fig. 2d). Next, to determine if the higher expression of NQO1-AS in MDA-LM2 relative to MDA-Par cells results in greater usage of the distal poly(A) site, we performed RNA-seq and 3′ end-seq in these cell lines^27^ (Fig. 2e). We found that MDA-LM2 cells had more RNA-seq reads 3′ of the proximal poly(A) site and there was increased use of the distal site in the 3′ end-seq data. These results support a model where HNRNPC binding, if not disrupted by NQO1-AS, favors the production of the *NQO1* isoform with a truncated 3′-UTR. To further test this model, we performed qPCR with reverse transcription (RT–qPCR) using isoform-specific primers in HNRNPC knockdown and control MDA-Par cells. On knockdown of HNRNPC, we observed an increase in overall NQO1 expression, as well as in the ratio of long-to-short *NQO1* isoforms (Fig. 2f,g). Knockdown of NQO1-AS had the opposite effect, decreasing the ratio of long-to-short isoforms (Fig. 2h). Conversely, overexpression of NQO1-AS increased the proportion of the long isoform (Fig. 2h). Finally, to confirm that this relationship between NQO1-AS expression and *NQO1* isoform ratios is not specific to the MDA-LM2 background, we measured the abundance of long and short *NQO1* isoforms in NQO1-AS knockdown BT-20 cells. As before, we found that depletion of NQO1-AS resulted in a decrease in the long-to-short isoform ratio (Fig. 2i). Together, these results suggest that HNRNPC binding to *NQO1* mRNA favors truncation of the 3′-UTR and that binding of NQO1-AS prevents this interaction and favors the full-length isoform.

### HNRNPA2B1 stabilizes the long NQO1 isoform

**Fig. 3:**
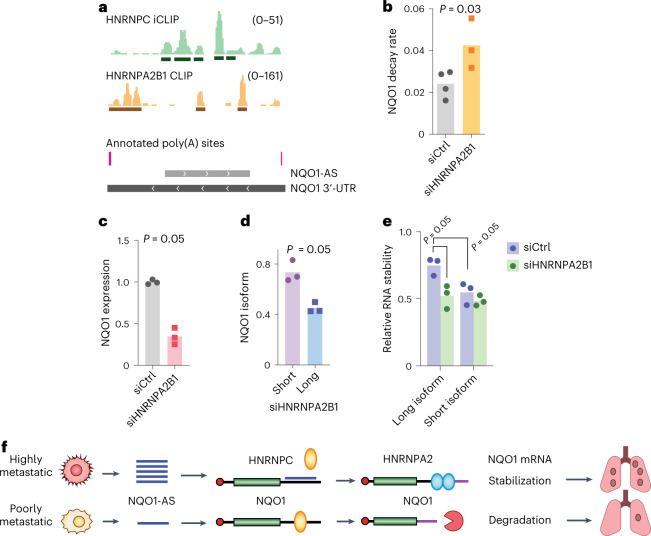
HNRNPA2B1 binds and stabilizes the long *NQO1* isoform and increases NQO1 expression. **a**, Read distribution in HNRNPC iCLIP and HNRNPA2B1 CLIP experiments relative to annotated poly(A) sites and NQO1-AS complementary region along the *NQO1* 3′-UTR. **b**, *NQO1* mRNA decay rate in MDA-LM2 cells with and without knockdown of HNRNPA2B1 (ref. ^21^). *n* = 4 independently treated cell cultures. **c**, RT–qPCR showing *NQO1* expression in MDA-LM2 cells with and without knockdown of HNRNPA2B1. *n* = 3 independent cell cultures. **d**, RT–qPCR showing *NQO1* long and short isoform fractions in HNRNPA2B1 knockdown MDA-LM2 cells, normalized by the same measurements in cells transfected with a nontargeting control. *n* = 3 independent cell cultures. **e**, Relative stability of long and short *NQO1* isoforms with and without HNRNPA2B1 knockdown in MDA-LM2 cells. *n* = 3 independent cell cultures. **f**, Summary of the mechanism by which overexpression of NQO1-AS causes NQO1 upregulation in highly metastatic breast cancer cells. All *P* values were calculated using a one-tailed Mann–Whitney *U*-test. Source data

**Extended Data Fig. 3:**
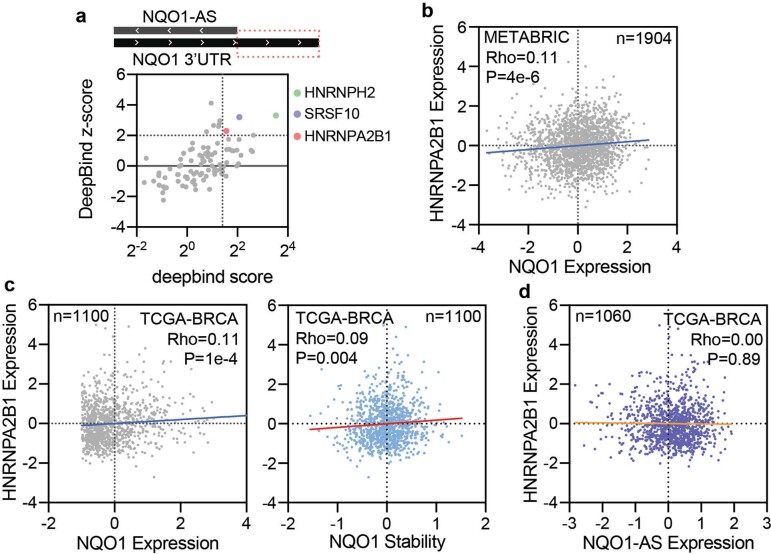
HNRNPA2B1 binds and stabilizes the long NQO1 isoform and increases NQO1 expression. (a) DeepBind sequence analysis of *NQO1* 3’UTR distal region complementary to NQO1-AS. Consensus motifs for HNRNPH2, SRSF10, and HNRNPA2B1 are highlighted. (b) Spearman correlation between NQO1 expression and HNRNPA2B1 expression in METABRIC dataset. N = 1904 patients. (c) Spearman correlation between NQO1 expression (left), *NQO1* stability (right) and HNRNPA2B1 expression in TCGA-BRCA dataset. N = 1100 patients. (d) Spearman correlation between NQO1-AS expression and HNRNPA2B1 expression in TCGA-BRCA dataset. N = 1060 patients. Source data

Based on our observation that NQO1-AS binding favors the long *NQO1* isoform and increases overall NQO1 expression, we hypothesized that the long isoform is more stable. To assess this, we analyzed the CLIP datasets to look for RBP binding sites that are present in the distal portion of the *NQO1* 3′-UTR^29^. This analysis revealed several binding sites for HNRNPA2B1, which we have previously shown to stabilize mRNA in MDA-MB-231 cells^21^ (Fig. 3a). Next, we performed an unbiased analysis of the distal 3′-UTR using DeepBind^30^ which revealed a strong match to the HNRNPA2B1 consensus motif (Extended Data Fig. 3a). Analyses of the Molecular Taxonomy of Breast Cancer International Consortium (METABRIC) and TCGA-BRCA datasets showed a positive correlation between HNRNPA2B1 and NQO1 expression (Extended Data Fig. 3b,c), and, in the case of the TCGA-BRCA dataset, between HNRNPA2B1 expression and *NQO1* stability (Extended Data Fig. 3c). These datasets did not show any correlation between HNRNPA2B1 and NQO1-AS expression (Extended Data Fig. 3d). On knockdown of HNRNPA2B1, we observed an increased *NQO1* mRNA decay rate and decreased expression (Fig. 3b,c). Notably, silencing of HNRNPA2B1 resulted in a greater decrease in the expression of the full-length *NQO1* transcript relative to the truncated isoform (Fig. 3d). Finally, separate measurement of the long and short *NQO1* isoform decay rates revealed that the long isoform was more stable and that this difference in stability was abrogated when HNRNPA2B1 was silenced (Fig. 3e). These results support the hypothesis that the two isoforms produced from alternate *NQO1* poly(A) site selection are differentially regulated and that HNRNPA2B1 is responsible for stabilizing the longer isoform.

Taken together, our results suggest that NQO1-AS overexpression in highly metastatic breast cancer cells decouples *NQO1* from the broader HNRNPC regulon, enabling NQO1 upregulation via a stabilizing interaction with HNRNPA2B1 (Fig. 3f).

### CTCF drives NQO1-AS transcription in highly metastatic cells

**Fig. 4:**
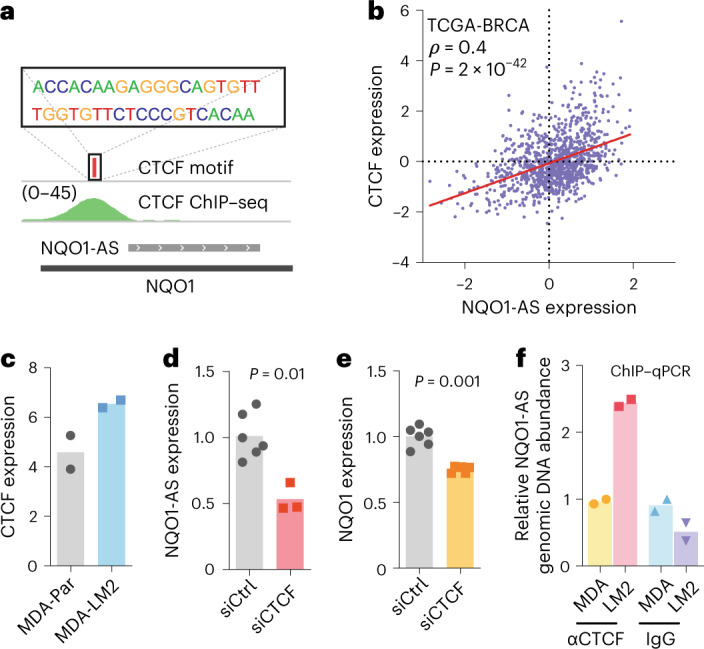
CTCF binding promotes NQO1-AS transcription in highly metastatic cells. **a**, CTCF consensus motif relative to CTCF ChIP–seq reads and NQO1-AS complementary region. **b**, Spearman correlation between CTCF expression and NQO1-AS expression in the TCGA-BRCA dataset. **c**, CTCF expression levels in MDA-Par and MDA-LM2 cells, as measured by RNA-seq. *n* = 2 independent cell cultures. **d**, qPCR showing NQO1-AS expression in MDA-LM2 cells with and without knockdown of CTCF. *n* = 6 independent cell cultures. **e**, qPCR showing *NQO1* expression in MDA-LM2 cells with and without CTCF knockdown. *n* = 6 independent cell cultures. **f**, ChIP–qPCR with precipitation of CTCF or IgG (control) in MDA-Par and MDA-LM2 cells. qPCR primers are targeting the NQO1-AS promoter region. *n* = 2 independent cell cultures. All *P* values were calculated using a one-tailed Mann–Whitney *U*-test. Source data

**Extended Data Fig. 4:**
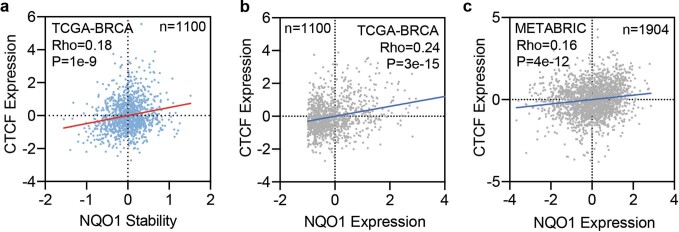
CTCF binding promotes NQO1-AS transcription in highly metastatic cells. (a) Spearman correlation between NQO1 stability and CTCF expression in TCGA-BRCA dataset. N = 1100 patients. (b) Spearman correlation between NQO1 expression and CTCF expression in TCGA-BRCA dataset. N = 1100 patients. (c) Spearman correlation between NQO1 expression and CTCF expression in METABRIC dataset. N = 1904 patients. Source data

We next sought to identify the factor(s) responsible for NQO1-AS upregulation in metastatic cells. Using DeepBind^30^ to perform an unbiased analysis of the putative NQO1-AS promoter, we discovered a strong match to the CTCF transcription factor consensus motif, with evidence of CTCF binding in multiple ChIP–seq datasets (Fig. 4a, ENCODE). We also found that CTCF expression was correlated with NQO1-AS in the TCGA breast cancer dataset (*ρ* = 0.4, *P* = 1 × 10^−16^; Fig. 4b). As CTCF is upregulated in highly metastatic MDA-LM2 cells (Fig. 4c), we hypothesized that CTCF drives NQO1-AS and, ultimately, NQO1 upregulation. Indeed, further analysis of the TCGA-BRCA and METABRIC datasets revealed a positive correlation between CTCF expression and *NQO1* stability and expression (Extended Data Fig. 4a–c). To test our hypothesis experimentally, we silenced CTCF in MDA-LM2 cells and observed a subsequent reduction in both NQO1-AS and NQO1 expression (Fig. 4d,e). We then performed ChIP qPCR to look for binding of CTCF to the NQO1-AS promoter in MDA-LM2 and MDA-Par cells. Consistent with the pattern of NQO1-AS expression, we saw significantly higher CTCF binding to the region upstream of NQO1-AS in the highly metastatic line (Fig. 4f).

### NQO1 and NQO1-AS promote metastatic lung colonization

**Fig. 5:**
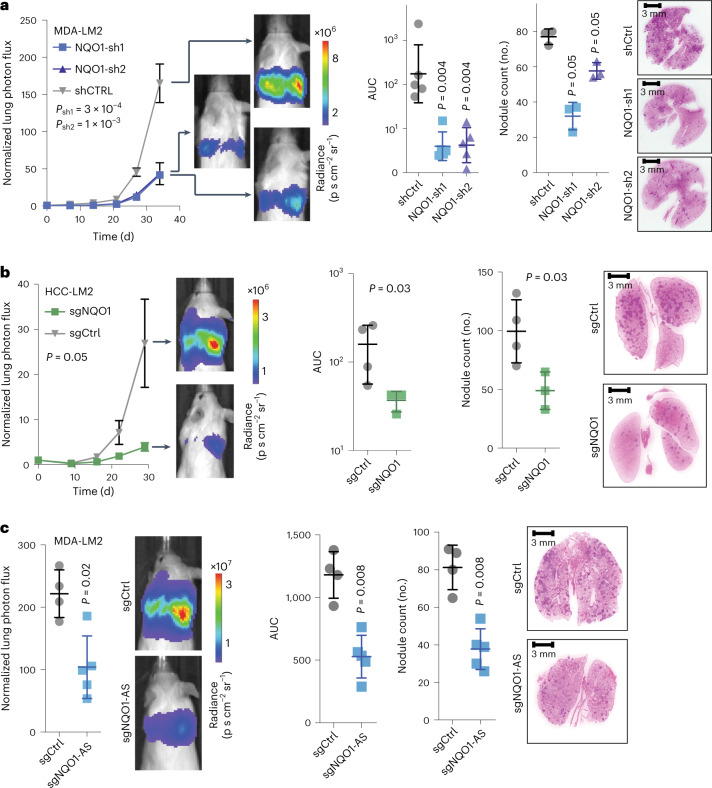
NQO1 and NQO1-AS promote metastatic lung colonization. **a**, Lung colonization assays comparing the in vivo metastatic colonization capacity of NQO1 knockdown and control MDA-LM2 cells. In vivo measurement of bioluminescence, by cancer cells that constitutively express luciferase, was used as a proxy for tumor burden and validated by endpoint lung histological sections. *n* = 5 mice per cohort. **b**, Lung colonization assays comparing the in vivo metastatic capacity of NQO1 knockdown and control HCC1806-LM2 cells. *n* = 4 mice per cohort. **c**, Lung colonization assays comparing the in vivo metastatic capacity of NQO1-AS knockdown and control MDA-LM2 cells. *n* = 4 mice per cohort. Data are presented as the mean ± s.e.m. All *P* values in the bioluminescence plots were calculated using a two-way analysis of variance (ANOVA). The *P* values in the area under the curve (AUC) and nodule count plots were calculated using a one-tailed Mann–Whitney *U*-test. Source data

**Extended Data Fig. 5:**
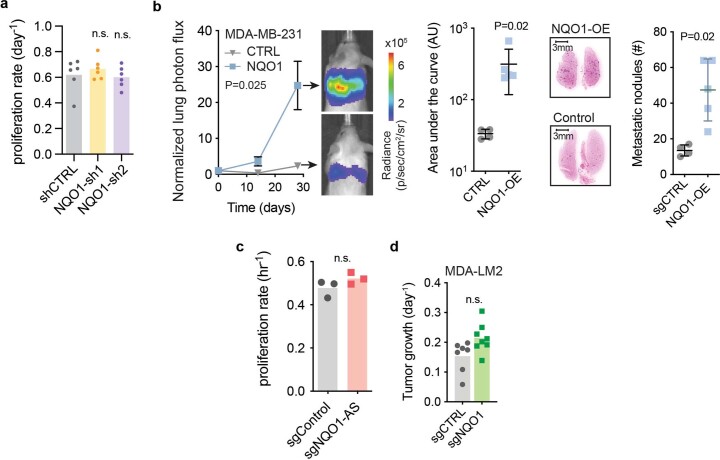
NQO1 and NQO1-AS promote metastatic lung colonization. (a) *in vitro* proliferation rate of control and shRNA-mediated NQO1 knockdown MDA-LM2 cells. N = 6 independent cell cultures. (b) *in vivo* lung colonization assay with MDA-Par control and NQO1 overexpression cells. Bioluminescence over time is shown on the left alongside representative images from each cohort at the end point. Total bioluminescence over the course of the experiment and nodule count at the end point are shown on the right, alongside representative H&E-stained lungs. Data are presented as mean ± SEM. N = 4 mice per cohort. (c) *in vitro* proliferation rate of control and CRISPRi-mediated NQO1-AS knockdown MDA-LM2 cells. N = 6 independent cell cultures. (d) *in vivo* primary tumor growth assay with control and NQO1 knockdown MDA-LM2 cells. N = 7 independent cell cultures. The *P* values in (a) were calculated using one-tailed Mann-Whitney U tests. The *P* values in (b) were calculated using two-way ANOVA for bioluminescence, and one-tailed Mann-Whitney U tests for AU and nodule counts. The *P* values in (c) and (d) were calculated using one-tailed Mann-Whitney U tests. Source data

We next investigated the impact of NQO1 upregulation on breast cancer progression. To assess this relationship experimentally, we performed in vivo lung colonization assays with NQO1 knockdown and control MDA-LM2 cells. While knocking down NQO1 did not affect the in vitro proliferation rate of cells (Extended Data Fig. 5a), it significantly decreased their capacity for lung colonization (Fig. 5a). We repeated this experiment in the HCC1806-LM2 breast cancer cell line^4^ and again observed a lower tumor burden in mice injected with NQO1 knockdown cells (Fig. 5b). Next, we performed lung colonization assays with MDA-Par cells overexpressing NQO1. We observed that mice injected with cells overexpressing NQO1 had significantly higher metastatic colonization capacity than control cells (Extended Data Fig. 5b). Given our previous results showing that NQO1-AS drives NQO1 upregulation, we expected to see similar results in lung colonization assays with NQO1-AS knockdown cells. Consistently, we found that NQO1-AS knockdown in MDA-LM2 cells resulted in decreased lung colonization capacity despite having no impact on the proliferation rate in vitro (Fig. 5c and Extended Data Fig. 5c). Together, our results suggest that increased expression of NQO1 and NQO1-AS in breast cancer cells promotes metastatic lung colonization.

We next asked whether NQO1 expression has a role in primary tumor growth. We injected NQO1 knockdown and control MDA-LM2 cells into mouse mammary fat pads and measured the tumors over time, observing no significant difference in growth rate between the two cohorts (Extended Data Fig. 5d). This result suggests that NQO1 does not have the same role in primary tumor growth as it does in lung colonization.

### NQO1 protects cancer cells from ferroptosis

**Fig. 6:**
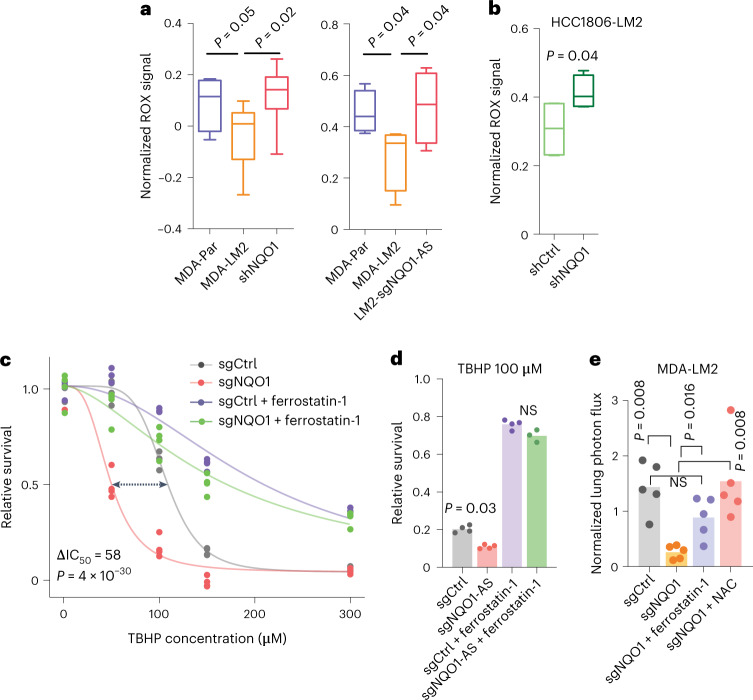
NQO1 protects cancer cells from oxidative stress and ferroptosis. **a**, ROS measured using the CellROX assay in MDA-Par, MDA-LM2, MDA-LM2 NQO1 knockdown and MDA-LM2 NQO1-AS knockdown cells. *n* = 4 independent cell cultures. **b**, ROS measured using the CellROX assay in HCC1806-LM2 NQO1 knockdown and control cells. *n* = 4 independent cell cultures. **c**, TBHP dose–response in MDA-LM2 NQO1 knockdown and control cells, with or without pretreatment with ferrostatin-1. *n* = 3 independently treated cell cultures. **d**, Relative survival of MDA-LM2 NQO1-AS knockdown and control cells after TBHP treatment, with and without pretreatment with ferrostatin-1. *n* = 4 independently treated cell cultures. **e**, In vivo lung colonization by MDA-LM2 control and NQO1 knockdown cells, with and without pretreatment with ferrostatin-1 or NAC, as measured by luciferase assay. *n* = 5 mice per cohort. In **a**,**b** the boxplots represent the median values and quartiles, and the whiskers represent the maximum and minimum values. The *P* values in **a**,**b** were calculated using an unpaired, one-tailed *t*-test. The *P* value in **c** was calculated using the drc package in R. In **d**,**e** *P* values were calculated using a one-tailed Mann–Whitney *U*-test. Source data

**Extended Data Fig. 6:**
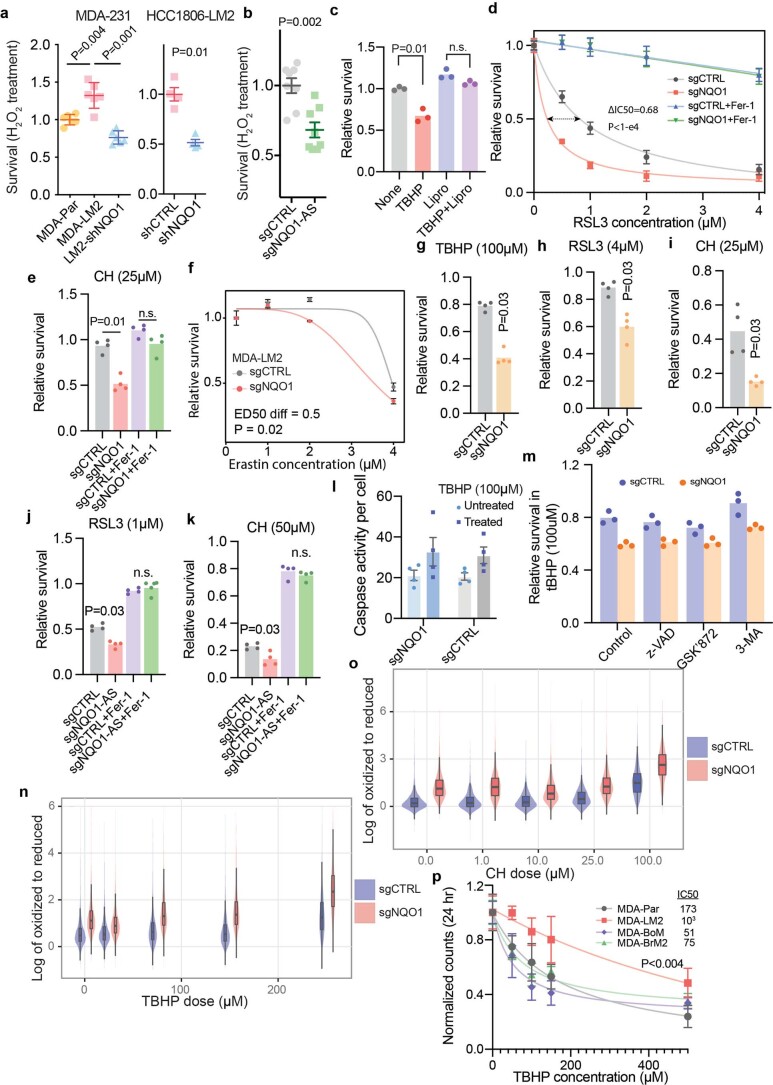
NQO1 protects cancer cells from ferroptosis. (a) Relative survival of MDA-Par, MDA-LM2, and shRNA-mediated NQO1 knockdown MDA-LM2 cells after treatment with H_2_O_2_ (N = 6 independently treated cell cultures), and relative survival of HCC1806-LM2 control and shRNA-mediated NQO1 knockdown cells after treatment with H_2_O_2_ (N = 4 independently treated cell cultures). (b) Relative survival of control and CRISPRi-mediated NQO1-AS knockdown MDA-LM2 cells after treatment with H_2_O_2_. N = 8 independently treated cell cultures. (c) Relative survival of MDA-LM2 NQO1 knockdown cells after TBHP treatment, with and without pretreatment with liproxstatin-1. N = 3 independently treated cell cultures. (d) RSL3 dose-response in MDA-LM2 NQO1 knockdown and control cells, with and without pretreatment with ferrostatin-1. N = 3 independently treated cell cultures. (e) Relative survival of MDA-LM2 NQO1 knockdown and control cells after cumene hydroperoxide treatment, with and without pretreatment with ferrostatin-1. N = 4 independently treated cell cultures. (f) Erastin dose-response in MDA-LM2 NQO1 knockdown and control cells. N = 3 independently treated cell cultures. (g) Relative survival of HCC1806-LM2 NQO1 knockdown and control cells after TBHP treatment. N = 4 independently treated cell cultures. (h) Relative survival of HCC1806-LM2 NQO1 knockdown and control cells after RSL3 treatment. N = 4 independently treated cell cultures. (i) Relative survival of HCC1806-LM2 NQO1 knockdown and control cells after cumene hydroperoxide treatment. N = 4 independently treated cell cultures. (j) Relative survival of MDA-LM2 NQO1-AS knockdown and control cells after RSL3 treatment, with and without ferrostatin-1 pretreatment. N = 4 independently treated cell cultures. (k) Relative survival of MDA-LM2 NQO1-AS knockdown and control cells after cumene hydroperoxide treatment, with and without pretreatment with ferrostatin-1. N = 4 independently treated cell cultures. (l) Relative caspase activity in TBHP treated and untreated NQO1 knockdown and control MDA-LM2 cells, measured using the Caspase Glo 3/7 Assay System from Promega. N = 4 independently treated cell cultures. (m) Relative survival of MDA-LM2 NQO1 knockdown and control cells after treatment with TBHP, with or without pretreatment with z-VAD, GSK’872, or 3-MDA. N = 3 independently treated cell cultures. (n) Lipid oxidation measurement in MDA-LM2 NQO1 knockdown and control cells after TBHP treatment and staining with C11-BODIPY dye. P < 10^-16^. N = 3 independently treated cell cultures. (o) Lipid oxidation measurement in MDA-LM2 NQO1 knockdown and control cells after cumene hydroperoxide treatment and staining with C11-BODIPY dye. P < 10^-16^. N = 3 independently treated cell cultures. (p) TBHP dose-response in MDA, MDA-LM2, MDA-BoM, and MDA-BrM2 cells. Data are presented as mean ± SD. N = 3 independently treated cell cultures. Data in (a), (b) and (l) are presented as mean ± SEM. Data in (d), (f), and (p) are represented as mean ± SD. Box plots in (n) and (o) represent median value and quartiles, whiskers represent 1.5 x IQR. The *P* values in (a), (b), (e), (g), (h), (i), (j), and (k) were calculated using one-tailed Mann-Whitney U tests. The *P* values in (c) were calculated using a two-tailed t-test. The *P* value in (d) was calculated using two-way ANOVA. The *P* value in (f) was calculated using the drc package in R. The *P* value in (p) was calculated using 2-way ANOVA. Source data

We next sought to determine the mechanism by which NQO1 exerts its pro-metastatic effects. NQO1 is a chemoprotective enzyme involved in cellular defense against oxidizing agents^31^. It reduces a wide range of substrates, counteracts the production of reactive oxygen species (ROS) and helps scavenge superoxides^32^. One of the most commonly mutated genes in non-small-cell lung cancer is nuclear factor erythroid 2-related factor 2 (*NRF2*), which drives oncogenic progression in this context in part by activating *NQO1* transcription, thereby increasing superoxide scavenging^33^. By overexpressing NQO1-AS, breast cancer cells may achieve the same result through a post-transcriptional mechanism. Additionally, it was recently shown that melanoma cells reversibly increase their expression of NADPH-generating enzymes to withstand oxidative stress^34^. NQO1 is directly involved in NADPH metabolism; thus, its upregulation in highly metastatic breast cancer cells may serve a similar purpose, given that breast cancer cells also experience oxidative stress during metastasis^35^. We investigated this possibility using NQO1 knockdown and control cells in both MDA-LM2 and HCC1806-LM2 backgrounds, and we observed significantly higher ROS in NQO1 knockdown cells in both backgrounds, as measured using a fluorometry assay (Fig. 6a,b). Additionally, we observed a significant decrease in the survival of NQO1 knockdown cells when treated with hydrogen peroxide (Extended Data Fig. 6a). MDA-Par cells, which have low endogenous NQO1 levels, also showed a lower tolerance to H_2_O_2_ treatment. We repeated these experiments in NQO1-AS knockdown MDA-LM2 cells and, consistent with the dependence of NQO1 levels on NQO1-AS, found that NQO1-AS knockdown cells had higher baseline ROS levels and increased sensitivity to H_2_O_2_ (Fig. 6a and Extended Data Fig. 6b).

Ferroptosis, a non-apoptotic form of regulated cell death, has recently been shown to be a consequence of excessive oxidative stress in metastatic breast cancer^36,37^. Given this, we hypothesized that NQO1 upregulation may be a mechanism for breast cancer cells to protect themselves from ferroptosis. To test this, we treated MDA-LM2 NQO1 knockdown and control cells with tert-butyl hydroperoxide (TBHP), a known inducer of oxidative stress and ferroptosis^38^. We found that the NQO1 knockdown cells were significantly more sensitive to TBHP (Fig. 6c). Furthermore, treatment with ferrostatin-1, an inhibitor of ferroptosis^39^, rescued TBHP-mediated toxicity, suggesting that the observed cell death was due, at least in part, to this mechanism. Importantly, while pretreatment with ferrostatin-1 did not entirely suppress TBHP-mediated cell death in this experiment, it negated the difference between NQO1 knockdown and control cells. We found that liproxstatin-1, another ferroptosis inhibitor^39^, could also rescue TBHP-mediated cell death in NQO1 knockdown cells (Extended Data Fig. 6c). We then tested the sensitivity of these cells to two additional ferroptosis inducers, RAS-selective lethal 3 (RSL3) (Extended Data Fig. 6d)^40^ and cumene hydroperoxide (CH) (Extended Data Fig. 6e), which induces lipid-specific oxidative damage. We found that NQO1 knockdown cells exhibited increased sensitivity to both of these compounds; in each case, this phenotype was rescued by ferrostatin-1 pretreatment. We then treated NQO1 knockdown and control cells with erastin, another ferroptosis-inducing agent, and once again found that cells deficient in NQO1 were more sensitive (Extended Data Fig. 6f). To confirm that these results were not specific to the MDA-LM2 background, we repeated sensitivity testing of HCC1806-LM2 cells to TBHP, RSL3 and CH; in each case, we found that NQO1 knockdown increased sensitivity (Extended Data Fig. 6g–i). Next, we asked if knockdown of NQO1-AS was sufficient to cause increased sensitivity to ferroptosis. In the MDA-LM2 background, we found that NQO1-AS knockdown cells were significantly more sensitive to TBHP, RSL3 and CH than controls; once again, the toxicity of these compounds could be rescued by pretreatment with ferrostatin-1 (Fig. 6d and Extended Data Fig. 6j,k).

In addition to protecting against ferroptosis, NQO1 may also shield cells against other forms of cell death. To address the role of NQO1 in sensitivity to apoptosis, we assayed the caspase activities of treated and untreated NQO1 knockdown and control cells using a luminescence-based assay. While cells treated with TBHP showed increased caspase activity, this effect was independent of NQO1 knockdown (Extended Data Fig. 6l). We next treated NQO1 knockdown and control cells with z-VAD, a pan-caspase inhibitor that blocks apoptosis^41^, GSK′872, a kinase inhibitor that blocks necroptosis^42^, and 3-methyladenosine (3-MA), an inhibitor of autophagy^43^, before treatment with TBHP (Extended Data Fig. 6m). We found that z-VAD and GSK′872 had no effect on TBHP toxicity, and that 3-MA pretreatment led to moderate rescue that was equivalent between NQO1 knockdown cells and controls. These results suggest that NQO1 does not protect against apoptosis, necroptosis or autophagy, and that its protective role in these assays has at least some specificity to ferroptosis. To further demonstrate the effect of NQO1 expression on sensitivity to ferroptosis, we stained for lipid oxidation in NQO1 knockdown and control cells using the BODIPY C11 dye after treatment with either TBHP (Extended Data Fig. 6n) or CH (Extended Data Fig. 6o). Under both treatment conditions, we observed significantly more lipid oxidation in the cells lacking NQO1.

Next, we asked if the resistance to oxidative stress that we observed in MDA-LM2 cells was also present in MDA-MB-231 cells that have been selected for metastasis to the bone (MDA-BoM) and brain (MDA-BrM2)^44^, which express NQO1 at a comparable level to MDA-Par cells^44^. We treated all four cell lines with TBHP and found that only the MDA-LM2 line showed significant resistance (Extended Data Fig. 6p). As the MDA-LM2 cell line was selected for metastasis to the lung, this result suggests that NQO1-mediated resistance to oxidative damage is unique to breast cancer metastases to the lung. Finally, we directly tested the role of NQO1 as a ferroptosis suppressor in vivo by conducting lung colonization assays in four cohorts of mice: one injected with MDA-LM2 control cells; one with untreated NQO1 knockdown cells; one with NQO1 knockdown cells pretreated with ferrostatin-1; and one with NQO1 knockdown cells pretreated with *N*-acetylcysteine (NAC), a compound that nonspecifically protects against oxidative damage. As before, we found that lung colonization by the NQO1 knockdown cells was significantly reduced. Notably, however, both ferrostatin-1 and NAC pretreatment rescued this defect, as we observed no significant difference between either of these cohorts and the controls (Fig. 6e). Because ferrostatin-1 is a specific inhibitor of ferroptosis, this result indicates that NQO1 facilitates lung colonization in part by suppressing ferroptosis. Moreover, the fact that cells pretreated with NAC showed only a modestly increased signal relative to cells pretreated with ferrostatin-1 indicates that ferroptosis protection is the dominant mechanism by which NQO1 facilitates lung colonization.

**Fig. 7:**
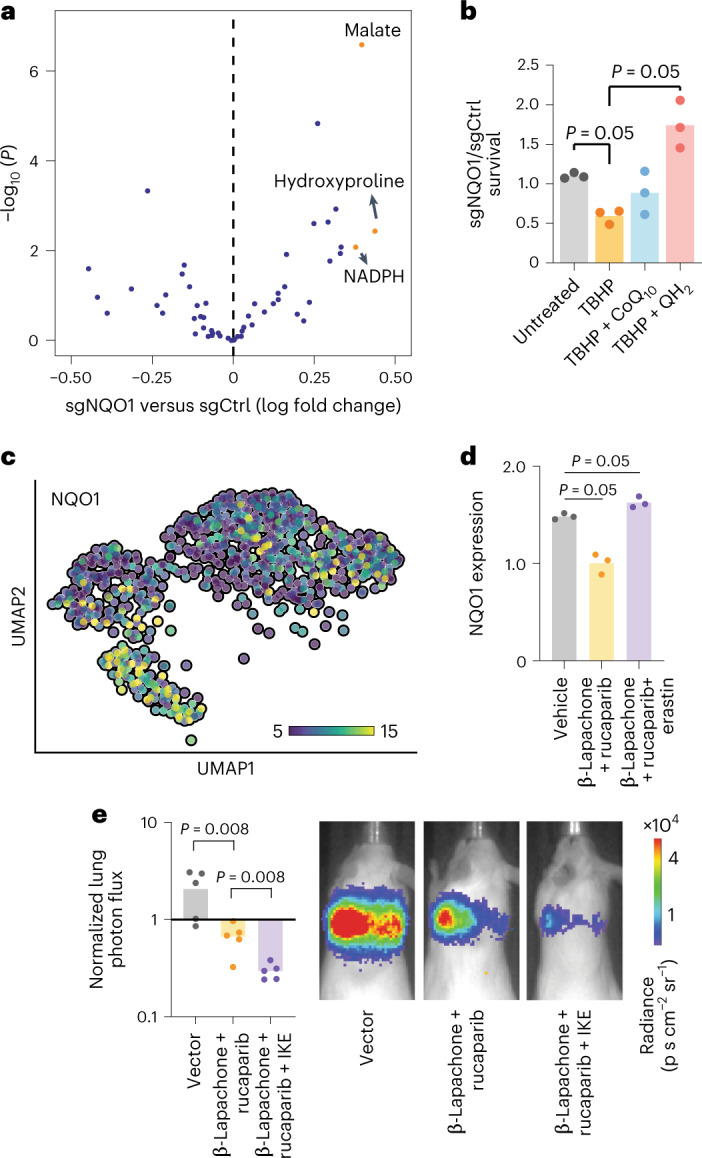
NQO1 mediates metabolic remodeling in breast cancer cells. **a**, Volcano plot showing the change in metabolic landscape of MDA-LM2 and HCC1806-LM2 cells in response to NQO1 knockdown. Metabolite levels were measured using LC–MS-based metabolic profiling. *n* = 3 independently treated cell cultures. **b**, Ratio of surviving NQO1 knockdown to control MDA-LM2 cells after pretreatment for 24 h with 10 µM ubiquinone (CoQ_10_) or ubiquinol (QH_2_), followed by treatment for 24 h with 150 µM TBHP. *n* = 3 independently treated cell cultures. **c**, UMAP plot generated from single-cell sequencing data in MDA-Par cells. Cells are colored according to relative NQO1 expression. **d**, qPCR showing the NQO1 expression level of MDA-LM2 cells after treatment with β-lapachone + rucaparib, β-lapachone + rucaparib + erastin or vector control. *n* = 3 independently treated cell cultures. **e**, Luciferase fluorescence signal from in vivo lung colonization assay with MDA-Par cells and subsequent treatment with β-lapachone + rucaparib, β-lapachone + rucaparib + IKE or vehicle control. Representative images from each cohort are shown. *n* = 5 mice per cohort. All *P* values were calculated using a one-tailed Mann–Whitney *U*-test. Source data

**Extended Data Fig. 7:**
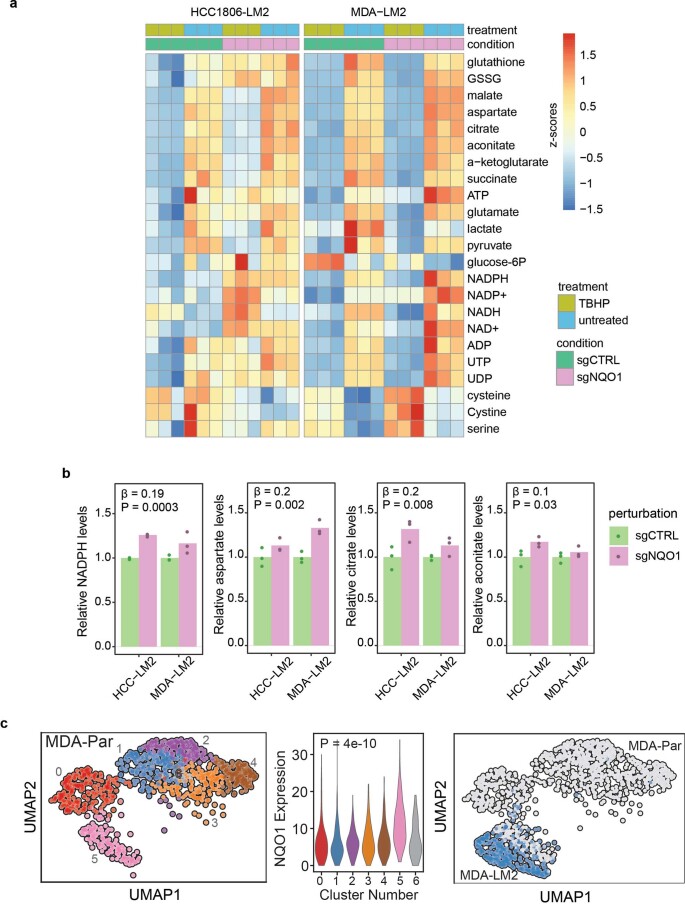
NQO1 mediates metabolic remodeling in cancer cells. (a) Heatmap showing metabolic changes in MDA-LM2 and HCC1806-LM2 NQO1 knockdown and control cells after treatment with TBHP. Metabolite levels were measured using LC/MS profiling. (b) Relative NADPH, aspartate, citrate, and aconitate levels in NQO1 knockdown and control MDA-LM2 and HCC1806-LM2 cells. N = 3 independently treated cell cultures. (c) UMAP visualization of single cell RNA-seq data from MDA-Par cells (left), NQO1 expression level by cluster (middle), and overlay with MDA-LM2 data (right). The *P* values in (b) were calculated using a linear model, with treatment status and genetic background as covariates. The *P* value in (c) was calculated using ANOVA. Source data

Because ferroptosis is associated with a disruption of normal cellular metabolism, we performed liquid chromatography (LC)–mass spectrometry (MS)-based metabolic profiling of breast cancer cells^45^. Consistent with the role of NQO1 as a regulator of the cell’s redox state, we observed a significant change in several redox-dependent metabolites on knockdown of NQO1 (Fig. 7a and Extended Data Fig. 7b). Specifically, NQO1 knockdown cells exhibited significantly higher NADPH, malate and hydroxyproline levels relative to controls (Fig. 7a). NQO1 coupled the reduction of ROS to the oxidation of NADPH; therefore, an increase in NADPH on knockdown of NQO1 (Extended Data Fig. 7b) is expected. To our knowledge, NQO1 is not directly involved in the metabolism of malate or hydroxyproline; however, the buildup of these metabolites in NQO1 knockdown cells may be due to a disruption in the metabolism of ubiquinone (coenzyme Q_10_ (CoQ_10_)). CoQ_10_ is converted to ubiquinol (QH_2_) by NQO1 and subsequently acts as an antioxidant^46^. CoQ_10_ is also used as an electron carrier by proline dehydrogenase 2, which catalyzes the first step of hydroxyproline catabolism^47^, and by the electron transport chain, of which malate dehydrogenase is an essential component. Therefore, decreased turnover of CoQ_10_ in the absence of NQO1 may perturb these metabolic pathways and cause the observed increase in hydroxyproline and malate. Ferroptosis suppressor protein 1 was recently found to suppress ferroptosis independently of GPX4 through the NADPH-dependent regeneration of CoQ_10_ from QH_2_ (refs. ^48,49^). To explore whether the impact of NQO1 on ferroptosis relates to CoQ_10_ metabolism, we pretreated NQO1 knockdown and control MDA-LM2 cells with either CoQ_10_ or QH_2_ and measured their sensitivity to TBHP. We found that pretreatment with QH_2_ rescued the increased sensitivity to TBHP previously observed in NQO1 knockdown cells (Fig. 7b). Pretreatment with CoQ_10_, however, resulted in a mild but statistically nonsignificant rescue of this phenotype. These results suggest that decreased NQO1 activity limits a cell’s ability to use CoQ_10_ as an antioxidant, and that NQO1 protects against ferroptosis at least in part through the production of QH_2_.

Next, we treated cells from both the MDA-LM2 and HCC1806-LM2 backgrounds with TBHP and repeated the metabolic profiling. As expected, TBHP treatment caused changes in many redox-dependent metabolites, which were relatively consistent across the two backgrounds (Extended Data Fig. 7a). This result highlights the extent of the metabolic remodeling that occurs with changes in NQO1 expression, facilitating breast cancer cell resistance to ferroptosis.

### The NQO1 and NQO1-AS pathway can be exploited therapeutically

Given the role of NQO1 in promoting breast cancer metastasis, we explored ways in which it could be targeted therapeutically. Previous work demonstrated that the compound β-lapachone is metabolized by NQO1 into an unstable hydroquinone that spontaneously generates superoxide^50^, leading to programmed necrosis of cancer cells^51^. This compound has been effective in cancers with increased NQO1 expression; however, its sustained use at high concentrations causes anemia in both human and animal models^52^. Combining β-lapachone with poly(ADP-ribose) polymerase (PARP) inhibitors results in synergistic antitumor activity, as DNA lesions caused by ROS cannot be repaired^53^. Given that NQO1 protects against ferroptosis, we hypothesized that low levels of imidazole ketone erastin (IKE), a ferroptosis-inducing agent with improved bioavailability^54^, could enhance the potency of β-lapachone and PARP inhibitor treatment. This hypothesis was in part inspired by the recent finding that inhibition of dihydroorotate dehydrogenase could sensitize cancer cells to ferroptosis on GPX4 inhibition, suggesting that therapeutics targeting ferroptosis-suppressing pathways in multiple places may act synergistically^55^. Additionally, single-cell RNA-seq (scRNA-seq) revealed a high degree of heterogeneity in NQO1 expression in MDA-Par cells (Fig. 7c). Cells expressing high levels of NQO1 clustered together in a uniform manifold approximation and projection (UMAP) analysis and overlapped with the cluster formed by MDA-LM2 cells when these cell lines were analyzed together (Extended Data Fig. 7c). This result suggests that the subpopulation of MDA-Par cells that express high levels of NQO1 are the precursors to the MDA-LM2 derivatives and are therefore an important subset to target therapeutically. Moreover, targeting PARP and NQO1 while inducing ferroptosis could simultaneously kill subpopulations of cancer cells with high and low NQO1 expression. To test this, we first treated MDA-LM2 cells with either β-lapachone + rucaparib (a PARP inhibitor), erastin or vehicle control, and then measured NQO1 expression in the surviving cells (Fig. 7d). We found that the cells that survived β-lapachone + rucaparib treatment had relatively low NQO1, whereas cells that survived erastin treatment had relatively high NQO1, indicating that these treatments indeed targeted distinct subpopulations. We then performed a lung colonization assay with MDA-Par cells in three cohorts of mice: one receiving β-lapachone + rucaparib; one receiving β-lapachone + rucaparib + IKE; and one receiving vehicle control. As we hypothesized, mice receiving β-lapachone, rucaparib and IKE had a significantly lower metastatic burden, as measured by in vivo bioluminescence (Fig. 7e). This result suggests that the role of NQO1 as a protective agent against ferroptosis can be exploited by treatment with an inducer of ferroptosis in combination with established NQO1 inhibitor therapies.

### NQO1-AS and NQO1 are associated with clinical metastasis

**Fig. 8:**
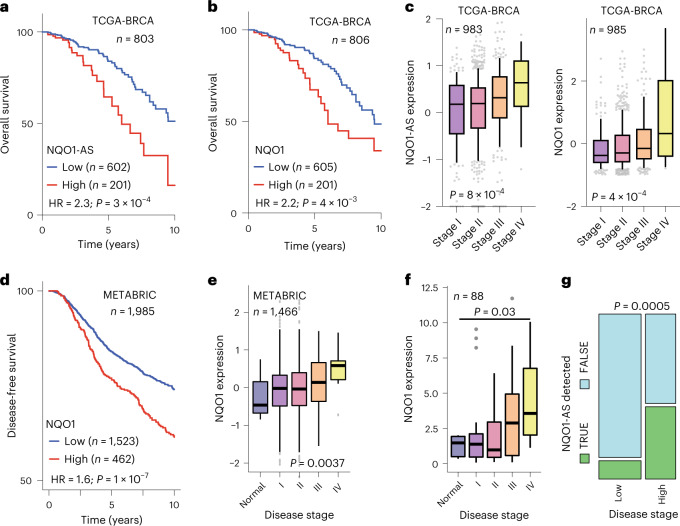
NQO1-AS and NQO1 expression are associated with metastasis in clinical samples. **a**, Kaplan–Meier curve showing the survival rates of patients with tumors expressing high or low levels of NQO1-AS. Data are from the TCGA-BRCA cohort. *n* = 803 patients per group. **b**, Kaplan–Meier curve showing the survival rates of patients with tumors expressing high or low levels of NQO1. Data are from the TCGA-BRCA cohort. *n* = 806 patients per group. **c**, NQO1-AS and NQO1 expression in tumors associated with stages I, II, III and IV of breast cancer. Data are from the TCGA-BRCA cohort. *n* = 983 patients (NQO1-AS). *n* = 985 patients (NQO1). **d**, Kaplan–Meier curve showing the disease-free survival of patients with tumors expressing high or low levels of NQO1. Data are from METABRIC. *n* = 1,985 patients per group. **e**, NQO1 expression in normal cells and cells associated with stages I, II, III and IV of breast cancer. Data are from METABRIC. *n* = 1,466 patients per group. **f**, NQO1 expression in normal cells and cells associated with stages I, II, III and IV of breast cancer. Data are from BCRT102 and BCRT103. *n* = 88 independent tissue samples. **g**, Graph showing the fraction of tumors with detectable NQO1-AS in low-stage and high-stage breast cancer. The width of the bars is proportional to the number of samples that fall into each group. Data are from the Origene Tissue Scan (BCRT102 and BCRT103). *n* = 88 independent tissue samples. The boxplots in **c**,**e**,**f** represent the median values and quartiles, while the whiskers represent the deciles. The *P* values in **a**,**b**,**d** were calculated using log-rank tests. The *P* values in **c**,**e** were calculated using an ANOVA. The *P* value in **f** was calculated using a two-tailed Mann–Whitney *U*-test. The *P* value in **g** was calculated using a chi-squared test. Source data

**Extended Data Fig. 8:**
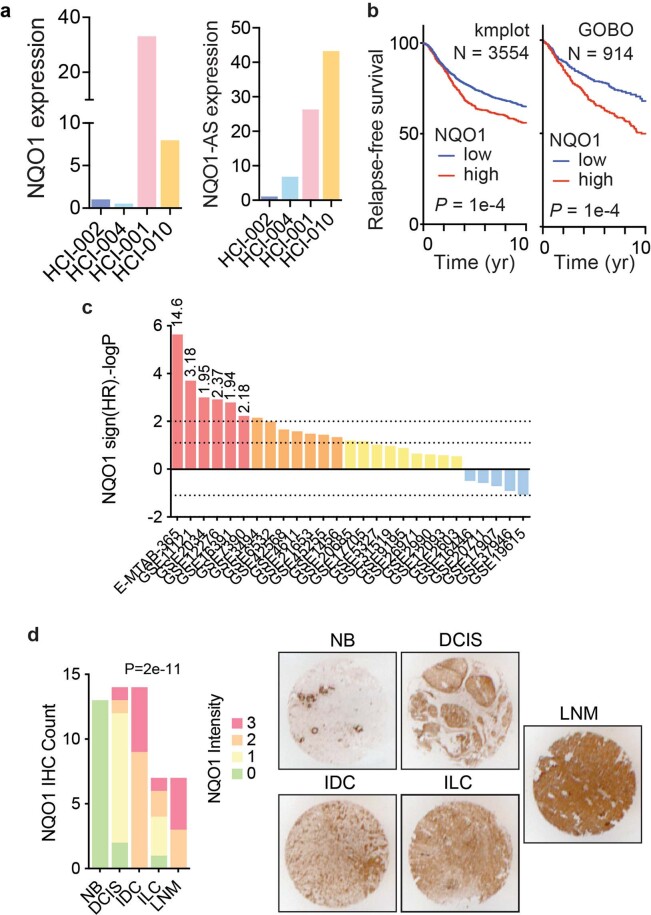
NQO1-AS and NQO1 expression are associated with metastasis in clinical samples. (a) Relative NQO1 (left) and NQO1-AS (right) expression in 4 patient-derived xenograft lines as measured by RNA-seq. (b) Kaplan-Meier curve showing relative relapse free survival of patients with tumors expressing high and low levels of NQO1 from kmplot (left, N = 3554 patients) and GOBO (right, N = 914 patients) databases. (c) Distribution of 10-year relapse-free survival *P* values (two-sided log rank test results reported as –logP for positive association and logP for negative) for the correlation of NQO1 expression and clinical outcome in the listed breast cancer datasets. Red bars show associations that pass the statistical threshold, orange bars are trending positive, and blue bars are trending negative. For statistically significant datasets, the hazard ratio is included at the top of the bar. (d) Immunohistochemical staining of NQO1 in a tissue microarray containing non-neoplastic breast tissue (NB), ductal carcinoma in situ (DCIS), invasive ductal carcinoma (IDC), invasive lobular carcinoma (ILC), and lymph node metastases (LNM). The experiment was performed once using 56 sections across stages of breast cancer progression. Blinded grading of the stain intensity is represented by the bar graph on the left. Representative images of stained specimens 3 mm in diameter are shown on the right. The *P* values in (b) were calculated using log rank tests. The *P* value in (d) was calculated using a χ^2^ test. Source data

To assess the broader clinical relevance of NQO1 in breast cancer metastasis, we examined the relationship between NQO1 expression and cancer progression in clinical samples. We analyzed RNA-seq data from two poorly (HCI-002 and HCI-004) and two highly (HCI-001 and HCI-010) metastatic patient-derived xenograft cell lines^56^, and found that the highly metastatic lines expressed significantly higher levels of NQO1 and NQO1-AS (Extended Data Fig. 8a). Our analysis of the TCGA-BRCA dataset showed significantly lower survival for patients with tumors expressing high levels of either NQO1-AS or NQO1 (Fig. 8a,b), both of which were positively correlated with disease stage (Fig. 8c). Our analysis of the METABRIC dataset yielded similar results, with high NQO1 expression associated with lower disease-free survival and higher disease stage (Fig. 8d,e). A negative association between NQO1 expression and survival was also observed in multiple other breast cancer gene expression datasets (Extended Data Fig. 8b,c). We also assayed a panel of cDNA derived from breast cancer tissue (Origene) and found that expression of NQO1 and NQO1-AS was associated with disease stage (Fig. 8f,g). Finally, we performed immunohistochemistry to assess NQO1 expression in breast cancer tissue from progressive cancer stages. Consistently, we found that there was significantly higher NQO1 expression in lymph node metastases, invasive lobular carcinoma and invasive ductal carcinoma than in ductal carcinoma in situ or nonneoplastic breast tissue (Extended Data Fig. 8d). Together, these results indicate that our findings in established cell lines are corroborated by clinical samples.

## Discussion

We have developed an integrated experimental and computational method to identify expressed antisense RNAs and applied it to an established model of breast cancer metastasis to profile antisense species that are upregulated in metastatic cells. In this study, we focused our attention on one such antisense RNA, NQO1-AS, whose sense transcript is similarly associated with breast cancer progression. Taken together, our data support a model in which NQO1-AS binds to the 3′-UTR of *NQO1*, preventing the binding of HNRNPC and thereby favoring the use of the distal polyadenylation site. HNRNPA2B1 can then bind to the long 3′-UTR, stabilizing mRNA and increasing the level of the NQO1 enzyme. Breast cancer cells exploit this pathway during metastatic progression, enabling them to tolerate higher levels of oxidative stress, while cells that successfully colonize the lungs are dependent on this pathway for survival, making them vulnerable to therapies that target this pathway. In our preliminary experiments, we showed that combining NQO1 and PARP inhibitors with a ferroptosis-inducing agent can significantly reduce metastatic burden in vivo.

Antisense RNAs are emerging as an important class of regulatory molecules with a wide range of functional roles; it is clear that they can alter gene expression at every level^57^. The pathway we identified in this study represents a mechanism by which cancer cells can enhance their metastatic capacity by regulating gene expression post-transcriptionally. Although we focused on a single pathway, the pool of antisense RNAs we identified probably includes several molecules that regulate gene expression through direct RNA–RNA interactions. Further investigation of these interactions may lead to a better understanding of how cells transition from healthy to diseased states, revealing new therapeutic targets.

In addition to gene expression dysregulation, metabolic reprogramming is an essential step in tumorigenesis and cancer progression^58^. Since its discovery, ferroptosis has drawn considerable interest as a form of regulated cell death that can be induced in multiple treatment-resistant cancers^59^. Triple-negative breast cancer, which is characteristically resistant to targeted therapies, undergoes ferroptosis in response to GPX4 inhibition with erastin^60^. In this study, we showed that MDA-MB-231 cells are able to protect themselves from cell death induced by oxidative damage, including ferroptosis, by overexpressing NQO1 and altering their metabolic profile. This metabolic remodeling coincides with increasing metastatic capacity; importantly, it makes the metastatic cells that have colonized the lungs dependent on NQO1 upregulation. This dependence creates a therapeutic opportunity because NQO1 can be targeted with β-lapachone, ‘unmasking’ the sensitivity of cancer to erastin. The use of β-lapachone in humans has been limited by toxicity when taken at high doses, but its inclusion in a more potent combination of drugs may enable the dose to be lowered to a level that is tolerable to patients. More work is required to identify the optimal drug combination, but we have demonstrated the viability of this therapeutic strategy.

## Methods

### Ethical regulations

All animal experiments were performed under the supervision and approval of the Institutional Animal Care and Uses Committee (IACUC) and the University of California, San Francisco (UCSF) (protocol no. AN179718-03F).

### Cell lines and cell culture

All cells were cultured at 37 °C in a humidified incubator with 5% CO_2_. MDA-MB-231 (ATCC HTB-26), MDA-LM2 (gifted by J. Massague) and HEK 293T (ATCC CRL-3216) cells were grown in DMEM supplemented with 10% FCS, penicillin (100 U ml^−1^), streptomycin (100 μg ml^−1^) and amphotericin (1 μg ml^−1^). HCC1806 (ATCC CRL-2335) and HCC1806-LM2 (gifted by S. Tavazoie) cells were grown in Roswell Park Memorial Institute-1640 medium supplemented with 10% FCS, l-glutamine (2 mM), sodium pyruvate (1 mM), penicillin (100 U ml^−1^), streptomycin (100 μg ml^−1^) and amphotericin (1 μg ml^−1^). BT-20 cells (ATCC HTB-19) were grown in EMEM supplemented with 10% FCS, penicillin (100 U ml^−1^), streptomycin (100 μg ml^−1^) and amphotericin (1 μg ml^−1^).

### Stable and transfected cell lines

Cells were transduced using the ViraSafe Lentiviral Packaging System (Cell Biolabs). The dCas9–KRAB and SunTag–VP64 systems were used for CRISPRi and CRISPRa, respectively, as described previously^61^. NQO1 was silenced both with short hairpin RNA (shRNA) and with CRISPRi constructs. NQO1-AS and HNRNPC were similarly silenced using CRISPRi. CTCF and HNRNPA2B1 were silenced using small interfering RNA (siRNA). NQO1 and NQO1-AS were overexpressed using CRISPRa. The shRNA and guide RNA sequences are shown in Supplementary Table 1.

### RT–qPCR

Transcript levels were measured using RT–qPCR by reverse transcribing total RNA into cDNA (SuperScript III or Maxima H Minus, Invitrogen), then using the PerfeCTa SYBR Green SuperMix (Quantabio) for amplification according to the manufacturer’s instructions. HPRT1 was used as the endogenous control. All primer sequences are shown in Supplementary Table 2. For NQO1-AS, we used a sequence-specific reverse transcription primer close to its 3′ end (AAGACTGAATCTACCTGCCCTAAG) to perform a strand-specific reverse transcription reaction.

### Rapid amplification of cDNA ends (5′ and 3′ RACE)

Total RNA was extracted from MDA-LM2 cells with the Zymo Quick-RNA Microprep Kit (Zymo Research). Poly(A) tailing of the isolated RNA was performed with yeast Poly(A) Polymerase (Jena Bioscience), allowing the capture of non-polyadenylated NQO1 antisense RNA in the following reverse transcription. First-strand cDNA synthesis was performed using the SMARTer Pico PCR cDNA Synthesis Kit (Takara Bio). The template switching mechanism enabled the generation of full-length cDNA as the template for downstream 5′ and 3′ RACE. PCR amplicons were generated using the primers shown in Supplementary Table 3, and then subjected to an additional round of PCR to add Illumina sequencing adapters. All the PCR reactions were done using SeqAmp DNA Polymerase (Takara Bio). The resulting libraries were then sequenced on an Illumina HiSeq 4000 sequencer.

### smRNA-FISH

MDA-LM2 cells were seeded on a coverslip coated with poly-d-lysine (0.1 mg ml^−1^) and cultured overnight in complete medium. Cells were fixed with 4% (w/v) methanol-free formaldehyde at room temperature for 10 min, followed by quenching with 0.1 M glycine solution at room temperature for 10 min. After several PBS washes, cells were permeabilized with 70% (v/v) ethanol overnight at 4 °C. Cells were then washed with the wash buffer (2× saline-sodium citrate (SSC) and 10% formamide) at room temperature for 5 min, followed by overnight incubation at 37 °C with hybridization buffer (2× SSC, 10% dextran sulfate, 10% formamide) mixed with each probe set at a final concentration of 0.125 µM. The following day, cells were washed twice with the wash buffer at 37 °C for 30 min. After a brief wash with 2× SSC buffer, coverslips were mounted on a SuperFrost Plus glass using ProLong Diamond antifade mountant with 4′,6-diamidino-2-phenylindole. Cells were imaged using the GE OMX-SR microscope in Z-stacks. Deconvolved images were imported into Fiji or ImageJ; the package RS-FISH^62^ was used to quantify smRNA-FISH spots. Fluorescent DNA probes (Quasar 570 for the NQO1 sense transcript and Quasar 670 for the antisense transcript, respectively) used in this experiment were synthesized by LGC Biosearch Technologies (Supplementary Table 4).

### RNA-seq library preparation

RNA-seq libraries were prepared using the ScriptSeq v2 Kit (Illumina) using rRNA-deleted RNA using the Ribo-Zero Gold Kit (Illumina). Libraries were sequenced on an Illumina HiSeq 4000 instrument at the UCSF Center for Advanced Technologies.

### 3′ end-seq

RNA-seq libraries were constructed with the QuantSeq REV Kit (Lexogen) according to the manufacturer’s protocol and sequenced on an Illumina HiSeq sequencer at the Center for Advanced Technology (UCSF). Cutadapt was used to remove short and low-quality reads, which were then aligned to the hg38 reference genome using salmon and compared using DESeq2 (ref. ^63^). Reads mapping to the annotated NQO1 poly(A) sites were extracted and compared between conditions using logistic regression.

### scRNA-seq

MDA-Par cells were split into biological replicates and barcoded; scRNA-seq libraries were prepared with the Chromium Next GEM Single Cell 3′ Kit v3 (10X Genomics). Libraries were sequenced on an Illumina NovaSeq sequencer at the Chan Zuckerberg Biohub.

### scRNA-seq analysis

#### Raw data processing

CellRanger v.3.0 (10X Genomics) was used for cell barcode filtering, read alignment, unique molecular identifier counting and to generate a digital gene expression matrix from raw FASTQ files. Reads were aligned to the human reference genome hg38 using CellRanger-provided annotations for gene features. Reads were assigned to cells based on their cell barcodes; barcodes that did not appear in the 10X Genomics 3M barcode allow list were removed.

#### Barcode demultiplexing and assignment

Cells were assigned to cell lines of origin by quantifying the relative proportion of detected genetic barcodes. Unique molecular counts for each barcode were determined from barcode-containing reads; a Gaussian kernel density estimation was fitted to the frequency of each barcode across all cells. The interpeak minima of the resulting bimodal distributions were set as the minimum threshold for barcode assignment. Cells were assigned a barcode if the frequency of that barcode exceeded its associated threshold and was tenfold more frequent than the second most frequently occurring barcode. Cells assigned multiple barcodes were designated as doublets and removed.

#### Single-cell data preprocessing and visualization

Scanpy^64^ was used for all preprocessing. Cells expressing fewer than 200 genes or more than 6,000 genes, or if aggregate mitochondrial gene expression was greater than 9% of overall cell expression, were removed. Genes expressed in fewer than three cells were also removed. Gene expression counts were normalized to 100,000 counts per cell, log-transformed after adding a pseudocount and scaled across cells to unit variance and zero mean. The top 3,000 highly variable genes were determined and cells were projected to a lower dimensional representation via principal component analysis with this reduced feature set. UMAP, implemented in scanpy.tl.umap with standard parameters, was then applied to cells represented by the minimum number of principal components required to explain the observed variance. Clusters were generated using the Louvain algorithm as implemented in scanpy.tl.louvain.

### IRIS

The basis of IRIS is schematized in Extended Data Fig. 1. First, for each gene, we identified the isoform with the longest coding sequence as the representative of that gene. We used the resulting FASTA file of sense RNAs as a reference to map GRO-seq reads from MDA-Par cells (bowtie v.2.3.5). Read mapping to the antisense strand was also tabulated and counted in 500-nt increments with a 250-nt step. The enrichment of antisense reads in every 500-nt window was assessed using logistic regression (Extended Data Fig. 1a). For this, the ratio of reads mapping to the 500-nt window of interest to the rest of the transcript was compared between the two strands (log fold change in antisense to sense ratio (logASR)). Interestingly, a higher presence of reads on the reverse strand was taken as evidence of antisense transcription (logASR > 0.5 and FDR < 0.01; Extended Data Fig. 1b). Significant neighboring windows were then merged using BEDTools v.2.28.0. The beginning of the first read and the end of the last read were used to refine the two ends of the identified antisense RNA species; both logASR and FDR were recalculated and the same thresholds were applied. The transcriptomic coordinates of the resulting antisense annotations were converted to genomic coordinates. This process was repeated for a ‘background’ set of loci, which were selected for the absence of any antisense transcript enrichment (logASR approximately 0 and FDR > 0.5).

As an independent measure of antisense transcript activity, we asked whether there was evidence of POLR2A binding in or upstream of the antisense of RNA species (above background) based on ENCODE ChIP–seq data. For this, we downloaded narrowPeak bed files (pre-irreproducibility discovery rate) for all available samples (67 samples total). We used the background loci from above to generate a null distribution for the numbered samples showing a POLR2A signal at each locus. We used this distribution to perform outlier analysis on our annotated antisense RNA species. We selected those species that were at least one interquartile range (IQR) above the background median (that is, median + IQR; Extended Data Fig. 1c); 308 antisense loci passed this filter.

Finally, we used stranded RNA-seq to further validate antisense transcription at these loci. This analysis was performed similarly to GRO-seq by calculating the logASR and the associated *P* value and FDR; 262 loci with a logASR > 1 and FDR < 1 × 10^−5^ were defined as our final antisense RNA annotation. Of these 262 loci, 58 overlapped with annotated transcripts in GENCODE v.28 (including any class of RNA, such as known genes and long noncoding RNAs); 20 were specifically annotated as ‘antisense’ or ‘pseudogenes’.

### Other computational tools

Salmon v.0.14.1 was used to quantify RNA-seq data for both custom sequences or annotated human transcriptomes (GENCODE v.28). APAlog (https://github.com/goodarzilab/APAlog) was used to quantify alternative polyadenylation.

### GRO-seq assays

Gro-seq was performed as previously described^65,66^, with some adaptations. For each sample, nuclei from 1 × 10^7^ MDA-Par or MDA-LM2 cells were used. All steps were done on ice. Cells were collected by scraping and then resuspended in swelling buffer (10 mM Tris-HCl pH 7.5, 2 mM MgCl_2_, 3 mM CaCl_2_) for 5 min on ice, then spun at 500*g* for 5 min at 4 °C. Swelling buffer was aspirated and 10 ml lysis buffer (10 mM Tris-HCl pH 7.5, 2 mM MgCl_2_, 3 mM CaCl_2_, 0.5% IGEPAL CA-630, 10% glycerol) was added. Nuclei were then pelleted at 1000*g* for 5 min at 4 °C. Lysis buffer was aspirated and nuclei were resuspended in 1 ml freezing buffer (50 mM Tris-HCl pH 8.3, 40% glycerol, 5 mM MgCl_2_, 0.1 mM EDTA). Nuclei were then pelleted at 1,000*g* for 5 min at 4 °C. Freezing buffer was aspirated and nuclei were resuspended in 100 μl freezing buffer and stored at −80 °C. For the run-on assay, frozen nuclei were thawed on ice and mixed with 100 μl reaction buffer (10 mM Tris-HCl pH 8.0, 5 mM MgCl_2_, 1 mM dithiothreitol (DTT), 300 mM KCl, 0.2 U μl^−1^ SUPERase inhibitor, 1% sarkosyl, 500 μM each of ATP, GTP and 5-bromouridine-5′-triphosphate, and 2 μM CTP), then incubated at 30 °C for 5 min. The reaction was stopped by adding 600 μl TRIzol LS (Thermo Fisher Scientific) and RNA was isolated according to the manufacturer’s protocol. The RNA pellet was resuspended in 20 μl H_2_O and the RNA was fractionated by adding 5 μl of 1 M NaOH and was incubated on ice for 40 min. The reaction was stopped by adding 25 μl of 1 M Tris-HCl, pH 6.8. This solution was run through Micro Biospin P-30 columns (BioRad Laboratories), then DNase-treated by adding 10 μl RQ1 DNase (Promega Corporation), 6.7 μl 10× DNase reaction buffer and 1 μl SUPERase inhibitor, and then incubated at 37 °C for 10 min. RNA was dephosphorylated by adding 5 μl Antarctic phosphatase (New England Biolabs), 8.5 μl 10× phosphatase buffer and 1 μl SUPERase inhibitor, and incubated at 37 °C for 1 h. Labeled RNA was isolated with anti-bromodeoxyuridine agarose beads (Santa Cruz Biotechnology) that were prepared by incubating in blocking buffer (0.5× Saline-sodium phosphate-EDTA (SSPE) buffer, 1 mM EDTA, 0.05% Tween 20, 0.1% polyvinylpolypyrrolidone, 1 mg ml^−1^ BSA) for 1 h at 4 °C, then resuspended in 500 μl binding buffer (0.5× SSPE, 1 mM EDTA, 0.05% Tween 20). RNA was added to the prepared beads after it was heated at 65 °C for 5 min and then placed on ice. RNA was incubated with the beads with end-over-end rotation for 1 h at 4 °C. The beads were then washed once with low-salt buffer (0.2× SSPE, 1 mM EDTA, 0.05% Tween 20), two times with high-salt buffer (0.5× SSPE, 1 mM EDTA, 0.05% Tween 20, 150 mM NaCl) and two times with TE buffer (pH 7.4, 0.05% Tween 20). RNA was eluted by adding 125 μl elution buffer (5 mM Tris-HCl pH 7.5, 300 mM NaCl, 1 mM EDTA, 0.1% SDS, 20 mM DTT) and rotated end-over-end at room temperature. Elution was repeated four times. RNA was isolated from the eluate using acid phenol:chloroform extraction and precipitation. The RNA pellet was resuspended in 45 μl H_2_O and then phosphorylated by adding 5.2 μl T4 polynucleotide kinase (PNK) buffer, 1 μl SUPERase inhibitor and 1 μl T4 PNK (New England Biolabs), and incubated at 37 °C for 1 h. RNA was isolated from the reaction using acid phenol:chloroform extraction and precipitation. The RNA was poly(A)-tailed by adding 0.8 μl 10× poly(A) polymerase buffer, 1 μl of 1 mM ATP, 0.5 μl SUPERase inhibitor and 0.75 μl poly(A) polymerase (New England Biolabs), and incubated at 37 °C for 30 min. RNA was then reverse-transcribed by first adding 1 μl of 10 mM deoxynucleoside triphosphates (dNTPs) and 2.5 μl of 12.5 μM oNTI223 primer, and was incubated at 75 °C for 3 min, then on ice for 1 min; then 2 μl 10× SuperScript III buffer, 2 μl of 25 mM MgCl_2_, 3 μl of 0.1 M DTT, 0.5 μl SUPERase inhibitor and 1 μl SuperScript III were added and RNA was incubated at 48 °C for 20 min. Excess primer was digested by adding 4 μl exonuclease I and incubating at 37 °C for 1 h. RNA was hydrolyzed by adding 1.8 μl of 1 M NaOH and incubating at 98 °C for 20 min; then, 1.8 μl of 1 M HCl was added to neutralize the reaction. The resulting cDNA was run on a 10% polyacrylamide Tris-Borate and EDTA (TBE)-urea gel (Invitrogen); the region from 110 nt to 405 nt was excised and the DNA recovered using the crush soak method. The precipitated DNA was resuspended in 7.5 μl H_2_O and circularized by adding 1 μl CircLigase buffer (Lucigen), 0.5 μl of 1 mM ATP, 0.5 mM MnCl_2_ and 0.5 μl CircLigase, and incubated at 60 °C for 1 h, then at 80 °C for 20 min. Linearization was performed by adding 3.8 μl of 100 mM KCl, 2 mm DTT and 1.5 μl APE1 (New England Biolabs), followed by incubation at 37 °C for 1 h. The cDNA was run on a 10% polyacrylamide TBE-urea gel and the region from 125 nt to 305 nt was excised; the DNA was recovered using the crush soak method. The precipitated cDNA was resuspended in 20 μl H_2_O. PCR was then performed by combining 10 μl of the cDNA with 4 μl 5× Phusion HF buffer, 0.4 μl of 10 mM dNTPs, 2 μl each of 5 μM oNTI200 and oNT1201, 1.4 μl H_2_O and 0.2 μl Phusion HF DNA polymerase (New England Biolabs), and running the following cycle: 98 °C for 30 s, then repeat 13 cycles of 98 °C for 10 s, 60 °C for 15 s and 72 °C for 15 s. The PCR product was run on an 8% polyacrylamide TBE gel and the region from 150 bp to 230 bp was excised. DNA was extracted using the crush soak method and the library was sequenced on an Illumina HiSeq 2000 system with the Illumina small RNA sequencing primer.

### Psoralen crosslinking followed by nuclease digestion and RNA ligation

MDA-Par and MDA-LM2 cells were resuspended in 4 ml ice-cold aminomethyltrioxsalen solution (0.5 mg ml^−1^ in PBS) and incubated on ice for 15 min in the dark. The mixture was then transferred to a 10-cm prechilled tissue culture plate and irradiated with 400 mJ cm^−^^2^ 254 nm ultraviolet light for 7 min with mixing every 2 min. (The plate was placed 3–4 cm away from the bulb.) Irradiated cells were then transferred to cold tubes and spun at 330*g* for 4 min to pellet. Crosslinked RNA was isolated using TRIzol followed by two chloroform extractions and isopropanol precipitation, with the final pellet dissolved in 50 µl water. Purified RNA was fragmented in a 50-µl reaction containing 20–50 µg RNA in 1× fragmentation buffer (catalog no. AM8740, Thermo Fisher Scientific) and heated at 70 °C for 2 min, at which point 2 µl of stop solution was added. The fragmented RNA was then purified using acid phenol:chloroform and the final pellet was dissolved in 100 µl water. The purified RNA was dephosphorylated by adding 1 µl 10× CutSmart buffer and 1 µl recombinant shrimp alkaline phosphatase (New England Biolabs) to 8 µl RNA; it was incubated at 37 °C for 30 min, then inactivated at 65 °C for 10 min. RNA was then 5′-phosphorylated by adding 4 µl 10× CutSmart buffer, 2.5 µl of 100 mM DTT, 2.5 µl of 100 mM ATP, 2.5 µl RNasin inhibitor (Promega Corporation), 2.5 µl T4 PNK and 26 µl H_2_O. The reaction was incubated at 37 °C for 30 min and inactivated at 65 °C for 10 min. RNA was then purified using the Zymogen RNA clean-up kit (Zymo Research). A total of 50 µl purified RNA was then mixed with 5 µl 10× T4 RNA ligase buffer, 2.5 µl of 100 mM ATP, 2.5 µl RNasin, 3 µl T4 RNA ligase I (New England Biolabs) and 25 µl H_2_O. Reactions were incubated overnight at 16 °C. SuperScript III was used for cDNA synthesis; NQO1_3utr_RT was used for priming (Supplementary Table 2). Samples without reverse transcriptase were used as controls in the subsequent qPCR.

### CLIP followed by RT–qPCR

Biological replicates of NQO1-AS knockdown and control MDA-LM2 cells were crosslinked with 400 mJ cm^−^^2^ 254 nm ultraviolet light. Crosslinked cells were lysed on ice with lysis buffer (100 mM Tris pH 7.5, 1% SDS, 1 mM EDTA) supplemented with SUPERase inhibitor and 1× protease inhibitor. The lysate was then clarified by spinning at 14,000*g* at 4 °C for 10 min. The clarified lysate was transferred to protein A Dynabeads conjugated to anti-HNRNPC (5 µg antibody per confluent 15-cm plate of cells, catalog no. sc-32308, Santa Cruz Biotechnology) and rotated end-over-end at 4 °C for 2 h. The beads were then washed once with high-stringency wash buffer (15 mM Tris pH 7.5, 5 mM EDTA, 1% Triton X-100, 1% sodium deoxycholate, 0.001% SDS, 120 mM NaCl, 25 mM KCl), once with high-salt wash buffer (15 mM Tris pH 7.5, 5 mM EDTA, 1% Triton X-100, 1% sodium deoxycholate, 0.001% SDS, 1 M NaCl) and once with ×1 PBS. The immunoprecipitated protein–RNA complexes were then treated with proteinase K (Thermo Fisher Scientific) reaction buffer (100 mM Tris pH 7.5, 100 mM NaCl, 1 mM EDTA, 0.2% SDS) for 45 min at 55 °C with intermittent mixing (900 rpm for 15 s, then 45-s rest). RNA was then extracted with acid phenol:chloroform and ethanol precipitated overnight at −20 °C. The purified RNA was then used for RT–qPCR as described above.

### ChIP followed by RT–qPCR

Biological replicates of MDA-Par and MDA-LM2 cells were crosslinked with 1% paraformaldehyde (PFA) for 10 min at room temperature. Formaldehyde was quenched with 1 M glycine (200 mM final concentration). Cells were then washed with PBS, scraped from the plate and collected by centrifugation. Next, cells were lysed with ChIP lysis buffer (50 mM HEPES-KOH pH 7.5, 140 mM NaCl, 1 mM EDTA pH 8, 1% Triton X-100, 0.1% sodium deoxycholate, 0.1% SDS, 1× protease inhibitor). DNA was fragmented by sonication (10 cycles on high, 30 s on and 30 s off, Diagenode Bioruptor UCD-200). An aliquot of the fragmented DNA was removed for size analysis; the remainder of the sample was diluted tenfold in radioimmunoprecipitation assay buffer (50 mM Tris-HCl pH 8, 150 mM NaCl, 2 mM EDTA pH 8, 1% NP-40, 0.5% sodium deoxycholate, 0.1% SDS, 1× protease inhibitor) and transferred to protein A Dynabeads conjugated to anti-CTCF (2 μg antibody per 25 μg chromatin, catalog no. ab188408, Abcam) and rotated end-over-end at 4 °C for 3 h. The beads were then washed once in low-salt wash buffer (20 mM Tris-HCl pH 8, 150 mM NaCl, 0.1% SDS, 1% Triton X-100, 2 mM EDTA), once in high-salt wash buffer (20 mM Tris-HCl pH 8, 500 mM NaCl, 0.1% SDS, 1% Triton X-100, 2 mM EDTA) and once in LiCl wash buffer (10 mM Tris-HCl pH 8, 0.25 M LiCl, 1% NP-40, 1% sodium deoxycholate, 1 mM EDTA). The immunoprecipitated protein–DNA complexes were eluted in elution buffer (1% SDS, 100 mM NaHCO_3_), treated with 2 µl RNase A (Thermo Fisher Scientific) overnight at 65 °C, and treated with 2 µl proteinase K for 1 h at 60 °C. The DNA was purified using a PCR purification kit (zymogen) and used as a template for qPCR as described above.

### In vitro proliferation

In vitro cancer cell proliferation assays were performed by seeding 5 × 10^4^ cells on day 0 and then counting them in triplicate on days 3 and 5. The slope of the best-fit line between the log of cell counts and days is the reported proliferation rate: logNT = logN0 + *rt* where *t* is the time in days and *r* the proliferation rate per day).

### Animal studies

In all cases, 7–12-week-old age-matched female NOD-*scid* gamma (NSG) mice (strain no. 005557, The Jackson Laboratory) were used. Female animals were used exclusively in this study because breast cancer is a disease that predominantly affects females.

### Metastatic lung colonization

Metastatic lung colonization assays were performed by injecting cancer cells stably expressing luciferase into mice via tail vein (5 × 10^4^ to 2.5 × 10^5^ cells per mouse for MDA-Par and MDA-LM2 and 1 × 10^5^ cells per mouse for HCC1806-LM2 cells). For the lung colonization experiment conducted with ferrostatin-1 and NAC pretreatment, MDA-LM2 NQO1 knockdown cells were treated with 1 µM ferrostatin-1 or 5 mM NAC for 1 h before injection. In vivo bioluminescence was measured by retro-orbital injection of luciferin (PerkinElmer) followed by imaging with an IVIS instrument (PerkinElmer). At the endpoint, lungs were extracted, fixed with PFA and subjected to hematoxylin and eosin (H&E) staining.

### In vivo primary tumor growth

Orthotopic tumor growth assays were performed by injecting 2.5 × 10^5^ cells resuspended in 50 μl PBS mixed with 50 μl Matrigel into the mammary glands of female NSG mice using a 28-gauge needle. Tumor volume was assessed using calipers to measure tumor length (L) and width (W) every 2 d, and calculated using the formula *π*LW2 / 6. The experimental endpoint was reached once tumors reached a volume of 500 mm^3^. The maximal tumor size of 20 mm in any direction permitted by the UCSF IACUC was not exceeded in this study.

### ROS measurements

NQO1 knockdown and control MDA-LM2 and HCC1806-LM2 cells were seeded at a density of 5 × 10^5^ per well in 6-well plates. The following day, ROS were measured using the CellROX Green Kit (Thermo Fisher Scientific) according to the manufacturer’s instructions. Then, cells were returned to normal growth medium and allowed to recover in the incubator for 1 h. They were then treated with 2 mM TBHP (catalog no. 458139, Sigma-Aldrich) for 30 min and assayed again using the CellROX Green Kit.

### H_2_O_2_ sensitivity assays

NQO1 knockdown and control MDA-LM2 or HCC1806-LM2 cells were seeded at a density of 2 × 10^5^ per well in 6-well plates. The following day, cells were treated with either 0.5, 1 or 1.5 mM H_2_O_2_. Cells were counted after 24 h of treatment.

### TBHP, RSL3 and CH dose–response assays with ferrostatin-1 rescue

MDA-LM2 or HCC1806-LM2 cells were seeded at 5,000 cells per well in triplicate per condition in a white opaque 96-well plate (catalog no. 3917, Corning) with 1 µM ferrostatin-1 (catalog no. SML0583, Sigma-Aldrich) or the equivalent volume of dimethylsulfoxide (DMSO.) Twenty-four hours later, cells were treated with the indicated concentrations of either TBHP, RSL3 (catalog no. SML2234, Sigma-Aldrich) or CH (from the Image-iT Lipid Peroxidation Kit (catalog no. C10445, Thermo Fisher Scientific). Twenty-four hours later, cell viability was measured with the CellTiter-Glo 2.0 Assay (catalog no. G9243, Promega Corporation) with 1,000 ms integration time.

### Liproxstatin-1 rescue assay

A total of 5 × 10^3^ NQO1 knockdown and control MDA-LM2 cells were seeded in 96-well plates. The next day, cells were treated with 1 µM liproxstatin-1 or DMSO vehicle for 1 h followed by 100 μM TBHP. After 24 h, the number of viable cells was determined using the CellTiter-Glo Luminescent Cell Viability Assay (Promega Corporation) according to the manufacturer’s protocol.

### BODIPY staining

MDA-LM2 guide Ctrl (gCtrl) and guide NQO1 (gNQO1) cells were seeded at 5,000 cells per well in triplicate per condition in a 96-well plate. Twenty-four hours later, cells were treated with 200 μM TBHP, 100 μM CH or vehicle for 2 h at 37 °C. Then, 10 μM BOPIDY C11 dye was added to the cells and incubated at 37 °C for 30 min. Cells were washed three times with PBS and fluorescence was read at 581/591 nm and 488/510 nm.

### Caspase activity assay

NQO1 knockdown and control MDA-LM2 cells were seeded 4 × 10^3^ per well in a 96-well plate. The next day, cells were treated with 100 µM TBHP for 24 h and assayed with the Caspase-Glo 3/7 Assay System (Promega Corporation) according to the manufacturer’s protocol.

### z-VAD, GSK′872 and 3-MA rescue assay

MDA-LM2 gCtrl and gNQO1 cells were seeded at 5,000 cells per well in triplicate per condition in a white opaque 96-well plate. Twenty-four hours later, cells were treated with 10 µM z-VAD (catalog no. FMK001, R&D Systems), 10 µM GSK′872 (catalog no. 6492, Tocris) or vehicle control for 2 h or 5 mM 3-MA (catalog no. M9281-100MG, Sigma-Aldrich) for 30 min. Cells were then treated with 100 µM TBHP. Twenty-four hours later, cell viability was measured with the CellTiter-Glo 2.0 Assay with 1,000 ms integration time.

### Metabolomics

Specialized medium was used for cell culture in the metabolomics experiments to enable mass spectroscopic analysis of cellular metabolites. For each experiment, half of the cells were grown in ‘H_2_O medium’, which contained 1× DMEM powder (catalog no. L80677054, Thermo Fisher Scientific), 10% dialyzed FCS, 100 U ml^−1^ penicillin, 100 µg ml^−1^ streptomycin, 1 µg ml^−1^ amphotericin, 2 mM l-glutamine, 25 mM glucose and 40 mM NaHCO_3_ dissolved in dialyzed H_2_O. The other half of the cells were grown in ‘D_2_O media’, which contained the same components dissolved in 50% dialyzed H_2_O and 50% D_2_O. NQO1 knockdown and control MDA-LM2 or HCC1806-LM2 cells were seeded at a density of 2 × 10^5^ per well in 6-well plates. The following day, cells were treated with 50 µM TBHP or left untreated. Twenty-four hours after treatment, lysates were collected by adding 400 µl chilled extraction buffer (40:40:20 acetonitrile:methanol:water + 0.5% v/v formic acid) to each well, incubating for 20–40 s at room temperature and quenching with 44 µl neutralization buffer (15% NH_4_HCO_3_ in water). Lysates were transferred to prechilled 1.5-ml tubes and frozen at −80 °C. Subsequent metabolomic profiling was performed as published previously^45^. Metabolomic data were acquired using with the Xcalibur software (v.4.0, Thermo Fisher Scientific) and analyzed with El-MAVEN (v.0.7.0) and ProteoWizard (v.3.0.20315).

### In vitro combined drug treatment

A total of 1.5 × 10^5^ MDA-Par cells were seeded in 6-well plates and treated the following day with 3 µM erastin or DMSO vehicle. Twenty hours later, cells were treated with 15 µM rucaparib or DMSO vehicle. Two hours later, cells were treated with 1, 2, 3 or 4 µM β-lapachone or DMSO vehicle. RNA was then extracted with the Quick-RNA Microprep Kit; NQO1 mRNA levels were assayed by RT–qPCR as detailed above.

### In vivo combined drug treatment

For the in vivo drug treatment studies, 5 × 10^5^ MDA-Par cells were injected via tail vein. Mice were then injected intraperitoneally with rucaparib (15 mg kg^−1^, Sigma-Aldrich) or retro-orbitally with saline control and β-lapachone (22 mg kg^−1^, Sigma-Aldrich) or HPβCD vehicle (600 mg kg^−1^, Sigma-Aldrich). IKE (23 mg kg^−1^, catalog no. 27088, Cayman Chemical) or saline control was injected intraperitoneally in the appropriate cohorts. β-Lapachone, rucaparib and IKE injections were repeated daily for 5 d. Lungs were extracted at the endpoint and stained with H&E.

### Immunohistochemistry

Tissue microarrays were obtained from the University of Virginia Cooperative Human Tissue Network. After deparaffinization by incubation in two baths of xylene for 10 min each, slides were then rehydrated by sequential incubation with 100%, 95%, 80% and 60% ethanol for 5 min each. Slides were then rinsed with distilled water three times for 3 min each. Antigen retrieval was done by placing the slides in boiling Tris-EDTA buffer, pH 9.0, and allowed to sit for 35 min. Slides were then rinsed three times with 1× PBS for 3 min each and placed in 3% H_2_O_2_ for 10 min to quench endogenous peroxidase activity. Slides were rinsed three times with 1× PBS for 3 min each and then blocked with 400 µl of 5% milk, and diluted in 1× PBS with Tween 20 (PBST) at room temperature for 1 h. Slides were then incubated with 400 µl anti-NQO1 (catalog no. 11451-1-AP, Proteintech) at a dilution of 1:200 in 1× PBS overnight at 4 °C. The next morning, slides were rinsed three times with 1× PBST for 3 min each and then incubated with 400 µl biotinylated goat anti-rabbit IgG secondary antibody (catalog no. BA-1000, Vector Laboratories), and then diluted 1:200 in 1× PBST at room temperature for 30 min. Slides were then washed three times with 1× PBST for 5 min each. Staining was done with VECTASTAIN ABC-HRP Kit (peroxidase, goat IgG, catalog no. PK-4005, Vector Laboratories). The ABC reagent was prepared and left at room temperature for 30 min as according to the manufacturer’s instructions. Slides were incubated with 400 µl of the prepared ABC reagent at room temperature for 30 min and then washed three times with 1× PBST 5 min each. Slides were then incubated with ImmPACT DAB Peroxidase (HRP) Substrate (catalog no. SK-4105, Vector Laboratories) until developed; afterwards, it was washed in distilled H_2_O twice for 5 min each. Slides were dehydrated by sequential incubation in 60%, 80%, 95% and 100% ethanol for 5 min each and then incubated in two baths of xylene for 2 min each. Slides were air-dried and scanned.

### Statistics and reproducibility

Statistical tests for the results shown in each figure panel are described in the figure legends. Where possible, tests that do not assume a normal distribution (that is, Mann–Whitney *U*-test) or are robust to violations of the normality assumption (that is, a two-way ANOVA) were used. The number of samples in each group were chosen based on the expected variation in the data. For the animal studies, mice were distributed into cohorts with 4–5 mice per cohort, which in an NSG background is enough to observe a greater than twofold difference with 90% confidence^3,5^. No data were excluded from the analyses. All experiments were randomized with regard to group assignment of samples or animals. Investigators were blinded during measurement of cellular fluorescence in the dose–response and cell counting experiments, and in the bioluminescence animal experiments. Investigators were not blinded during collection of the transcriptomic data (that is, RNA-seq analysis, qPCR).

### Reporting summary

Further information on research design is available in the Nature Portfolio Reporting Summary linked to this article.

## Supplementary information

Reporting Summary

Supplementary Tables 1–4. Table 1: KD-OE oligonucleotides. Table 2: RT–qPCR primers. Table 3: RACE primers. Table 4: smFISH probes

## Data Availability

All sequencing data produced in this study have been deposited in the Gene Expression Omnibus (GEO) repository under accession no. GSE186641. The breast cancer data from the TCGA research network analyzed in this study are available at https://portal.gdc.cancer.gov/projects/TCGA-BRCA. Breast cancer data from the METABRIC dataset analyzed in this study are available at https://ega-archive.org/studies/EGAS00000000083. The CLIP data from the CLIPdb^19^ is available at http://clipdb.ncrnalab.org. The ENCODE datasets are available at https://www.encodeproject.org. Hg38 (https://www.ncbi.nlm.nih.gov/assembly/GCF_000001405.40) was used as the human genome reference sequence. Previously published datasets analyzed in this study are available under the following GEO accession nos.: GSE49649 (ref. ^3^), GSE63605 (ref. ^4^), GSE76488 (ref. ^7^), GSE77634 (ref. ^20^), GSE35800 (ref. ^21^), GSE45827 (ref. ^23^), GSE56010 (ref. ^24^), GSE186647 (ref. ^27^) and GSE66092 (ref. ^28^). The source data for Figs. 1d,f–h, 2f–i, 3b–e, 4d–f, 5a–c, 6a–e and 7b,d–e, and Extended Data Figs. 1h–l, 5a–d and 6a–m,p have been provided as source data files. Source data are provided with this paper.

## Source data

Source Data Fig. 1 Statistical source data.
Source Data Fig. 2 Statistical source data.
Source Data Fig. 3 Statistical source data.
Source Data Fig. 4 Statistical source data.
Source Data Fig. 5 Statistical source data.
Source Data Fig. 6 Statistical source data.
Source Data Fig. 7 Statistical source data.
Source Data Fig. 8 Statistical source data.
Source Data Extended Data Fig. 1 Statistical source data.
Source Data Extended Data Fig. 2 Statistical source data.
Source Data Extended Data Fig. 3 Statistical source data.
Source Data Extended Data Fig. 4 Statistical source data.
Source Data Extended Data Fig. 5 Statistical source data.
Source Data Extended Data Fig. 6 Statistical source data.
Source Data Extended Data Fig. 7 Statistical source data.
Source Data Extended Data Fig. 8 Statistical source data.

## Code availability

The custom code used for IRIS is available at GitHub (https://github.com/goodarzilab/IRIS).

## References

[1] Minn AJ (2005). Genes that mediate breast cancer metastasis to lung. Nature.

[2] van ’t Veer LJ (2002). Gene expression profiling predicts clinical outcome of breast cancer. Nature.

[3] Goodarzi H (2014). Metastasis-suppressor transcript destabilization through TARBP2 binding of mRNA hairpins. Nature.

[4] Goodarzi H (2015). Endogenous tRNA-derived fragments suppress breast cancer progression via YBX1 displacement. Cell.

[5] Goodarzi H (2016). Modulated expression of specific tRNAs drives gene expression and cancer progression. Cell.

[6] Vanharanta S (2014). Loss of the multifunctional RNA-binding protein RBM47 as a source of selectable metastatic traits in breast cancer. eLife.

[7] Fish L (2016). Muscleblind-like 1 suppresses breast cancer metastatic colonization and stabilizes metastasis suppressor transcripts. Genes Dev..

[8] Pencheva N, Tavazoie SF (2013). Control of metastatic progression by microRNA regulatory networks. Nat. Cell Biol..

[9] Lu Z (2016). RNA duplex map in living cells reveals higher-order transcriptome structure. Cell.

[10] Sharma E, Sterne-Weiler T, O’Hanlon D, Blencowe BJ (2016). Global mapping of human RNA-RNA interactions. Mol. Cell.

[11] Aw JG (2016). In vivo mapping of eukaryotic RNA interactomes reveals principles of higher-order organization and regulation. Mol. Cell.

[12] Ozsolak F (2010). Comprehensive polyadenylation site maps in yeast and human reveal pervasive alternative polyadenylation. Cell.

[13] Tufarelli C (2003). Transcription of antisense RNA leading to gene silencing and methylation as a novel cause of human genetic disease. Nat. Genet..

[14] Rinn JL (2007). Functional demarcation of active and silent chromatin domains in human *HOX* loci by noncoding RNAs. Cell.

[15] Faghihi MA (2008). Expression of a noncoding RNA is elevated in Alzheimer’s disease and drives rapid feed-forward regulation of β-secretase. Nat. Med..

[16] Balbin OA (2015). The landscape of antisense gene expression in human cancers. Genome Res..

[17] Tavazoie SF (2008). Endogenous human microRNAs that suppress breast cancer metastasis. Nature.

[18] Lu Z, Gong J, Zhang QC (2018). PARIS: Psoralen Analysis of RNA Interactions and Structures with high throughput and resolution. Methods Mol. Biol..

[19] Yang Y-T (2015). CLIPdb: a CLIP-seq database for protein-RNA interactions. BMC Genomics.

[20] Van Nostrand EL (2016). Robust transcriptome-wide discovery of RNA-binding protein binding sites with enhanced CLIP (eCLIP). Nat. Methods.

[21] Goodarzi H (2012). Systematic discovery of structural elements governing stability of mammalian messenger RNAs. Nature.

[22] König J (2010). iCLIP reveals the function of hnRNP particles in splicing at individual nucleotide resolution. Nat. Struct. Mol. Biol..

[23] Gruosso T (2016). Chronic oxidative stress promotes H2AX protein degradation and enhances chemosensitivity in breast cancer patients. EMBO Mol. Med..

[24] Liu N (2015). N6-methyladenosine-dependent RNA structural switches regulate RNA–protein interactions. Nature.

[25] Fischl H (2019). hnRNPC regulates cancer-specific alternative cleavage and polyadenylation profiles. Nucleic Acids Res..

[26] Gruber AJ (2016). A comprehensive analysis of 3′ end sequencing data sets reveals novel polyadenylation signals and the repressive role of heterogeneous ribonucleoprotein C on cleavage and polyadenylation.. Genome Res..

[27] Navickas, A. et al. An mRNA processing pathway suppresses metastasis by governing translational control from the nucleus. Preprint at *bioRxiv*10.1101/2021.10.04.463118 (2021). 10.1038/s41556-023-01141-9 PMC10264242 37156909

[28] Hwang H-W (2016). PAPERCLIP identifies microRNA targets and a role of CstF64/64tau in promoting non-canonical poly(a) site usage. Cell Rep..

[29] Zarnack K (2013). Direct competition between hnRNP C and U2AF65 protects the transcriptome from the exonization of Alu elements. Cell.

[30] Alipanahi B, Delong A, Weirauch MT, Frey BJ (2015). Predicting the sequence specificities of DNA- and RNA-binding proteins by deep learning. Nat. Biotechnol..

[31] Ross D (2004). Quinone reductases multitasking in the metabolic world. Drug Metab. Rev..

[32] Siegel D (2004). NAD(P)H:quinone oxidoreductase 1: role as a superoxide scavenger. Mol. Pharmacol..

[33] Torrente L (2020). Inhibition of TXNRD or SOD1 overcomes NRF2-mediated resistance to β-lapachone. Redox Biol..

[34] Piskounova E (2015). Oxidative stress inhibits distant metastasis by human melanoma cells. Nature.

[35] Alvarez SW (2017). NFS1 undergoes positive selection in lung tumours and protects cells from ferroptosis. Nature.

[36] Dixon SJ, Stockwell BR (2019). The hallmarks of ferroptosis. Annu. Rev. Cancer Biol..

[37] Bi J (2019). Metadherin enhances vulnerability of cancer cells to ferroptosis. Cell Death Dis..

[38] Wenz C (2018). t-BuOOH induces ferroptosis in human and murine cell lines. Arch. Toxicol..

[39] Zilka O (2017). On the mechanism of cytoprotection by ferrostatin-1 and liproxstatin-1 and the role of lipid peroxidation in ferroptotic cell death. ACS Cent. Sci..

[40] Sui X (2018). RSL3 drives ferroptosis through GPX4 inactivation and ROS production in colorectal cancer. Front. Pharmacol..

[41] Van Noorden CJ (2001). The history of Z-VAD-FMK, a tool for understanding the significance of caspase inhibition. Acta Histochem..

[42] Kaiser WJ (2013). Toll-like receptor 3-mediated necrosis via TRIF, RIP3, and MLKL. J. Biol. Chem..

[43] Wu Y-T (2010). Dual role of 3-methyladenine in modulation of autophagy via different temporal patterns of inhibition on class I and III phosphoinositide 3-kinase. J. Biol. Chem..

[44] Bos PD (2009). Genes that mediate breast cancer metastasis to the brain. Nature.

[45] Bajad SU (2006). Separation and quantitation of water soluble cellular metabolites by hydrophilic interaction chromatography-tandem mass spectrometry. J. Chromatogr. A.

[46] Ross D, Siegel D (2017). Functions of NQO1 in cellular protection and CoQ_10_ metabolism and its potential role as a redox sensitive molecular switch.. Front. Physiol..

[47] Summitt CB (2015). Proline dehydrogenase 2 (PRODH2) is a hydroxyproline dehydrogenase (HYPDH) and molecular target for treating primary hyperoxaluria. Biochem. J..

[48] Doll S (2019). FSP1 is a glutathione-independent ferroptosis suppressor. Nature.

[49] Bersuker K (2019). The CoQ oxidoreductase FSP1 acts parallel to GPX4 to inhibit ferroptosis. Nature.

[50] Reinicke KE (2005). Development of β-lapachone prodrugs for therapy against human cancer cells with elevated NAD(P)H:quinone oxidoreductase 1 levels. Clin. Cancer Res..

[51] Huang X (2012). An NQO1 substrate with potent antitumor activity that selectively kills by PARP1-induced programmed necrosis. Cancer Res..

[52] Noh J-Y (2010). A naphthoquinone derivative can induce anemia through phosphatidylserine exposure-mediated erythrophagocytosis. J. Pharmacol. Exp. Ther..

[53] Huang X (2016). Leveraging an NQO1 bioactivatable drug for tumor-selective use of poly (ADP-ribose) polymerase inhibitors. Cancer Cell.

[54] Larraufie M-H (2015). Incorporation of metabolically stable ketones into a small molecule probe to increase potency and water solubility. Bioorg. Med. Chem. Lett..

[55] Mao C (2021). DHODH-mediated ferroptosis defence is a targetable vulnerability in cancer. Nature.

[56] DeRose YS (2011). Tumor grafts derived from women with breast cancer authentically reflect tumor pathology, growth, metastasis and disease outcomes. Nat. Med..

[57] Zhao S, Zhang X, Chen S, Zhang S (2020). Natural antisense transcripts in the biological hallmarks of cancer: powerful regulators hidden in the dark. J. Exp. Clin. Cancer Res..

[58] Pavlova NN, Thompson CB (2016). The emerging hallmarks of cancer metabolism. Cell Metab..

[59] Viswanathan VS (2017). Dependency of a therapy-resistant state of cancer cells on a lipid peroxidase pathway. Nature.

[60] Yu M (2019). Targeted exosome-encapsulated erastin induced ferroptosis in triple negative breast cancer cells. Cancer Sci..

[61] Horlbeck MA (2016). Compact and highly active next-generation libraries for CRISPR-mediated gene repression and activation. eLife.

[62] Bahry E (2022). RS-FISH: precise, interactive, fast, and scalable FISH spot detection. Nat. Methods.

[63] Love MI, Huber W, Anders S (2014). Moderated estimation of fold change and dispersion for RNA-seq data with DESeq2. Genome Biol..

[64] Wolf FA, Angerer P, Theis FJ (2018). SCANPY: large-scale single-cell gene expression data analysis. Genome Biol..

[65] Core LJ, Waterfall JJ, Lis JT (2008). Nascent RNA sequencing reveals widespread pausing and divergent initiation at human promoters. Science.

[66] Wang D (2011). Reprogramming transcription by distinct classes of enhancers functionally defined by eRNA. Nature.

