# Supplementary material for: A sense-antisense RNA interaction promotes breast cancer metastasis via regulation of NQO1 expression
Source: Nat Cancer. 2023 May 11;4(5):682–98. doi: 10.1038/s43018-023-00554-7 (PMC10212767; doi:10.1038/s43018-023-00554-7)
Supplement: Supplementary file 1 — Reporting Summary [file 43018_2023_554_MOESM1_ESM.pdf]

nature portfolio

Corresponding author(s): Hani Goodarzi

Last updated by author(s): Mar 24, 2023

Reporting Summary

Nature Portfolio wishes to improve the reproducibility of the work that we publish. This form provides structure for consistency and transparency in reporting. For further information on Nature Portfolio policies, see our Editorial Policies and the Editorial Policy Checklist.

Statistics

For all statistical analyses, confirm that the following items are present in the figure legend, table legend, main text, or Methods section.

n/a	Confirmed	
☐	☒	The exact sample size (*n*) for each experimental group/condition, given as a discrete number and unit of measurement
☐	☒	A statement on whether measurements were taken from distinct samples or whether the same sample was measured repeatedly
☐	☒	The statistical test(s) used AND whether they are one- or two-sided<br>*Only common tests should be described solely by name; describe more complex techniques in the Methods section.*
☐	☒	A description of all covariates tested
☐	☒	A description of any assumptions or corrections, such as tests of normality and adjustment for multiple comparisons
☐	☒	A full description of the statistical parameters including central tendency (e.g. means) or other basic estimates (e.g. regression coefficient) AND variation (e.g. standard deviation) or associated estimates of uncertainty (e.g. confidence intervals)
☐	☒	For null hypothesis testing, the test statistic (e.g. *F*, *t*, *r*) with confidence intervals, effect sizes, degrees of freedom and *P* value noted<br>*Give P values as exact values whenever suitable.*
☐	☒	For Bayesian analysis, information on the choice of priors and Markov chain Monte Carlo settings
☐	☒	For hierarchical and complex designs, identification of the appropriate level for tests and full reporting of outcomes
☐	☒	Estimates of effect sizes (e.g. Cohen's *d*, Pearson's *r*), indicating how they were calculated

Our web collection on statistics for biologists contains articles on many of the points above.

Software and code

Policy information about availability of computer code

Data collection	Thermo Fisher Scientific Xcalibur™ Software (4.0) was used for acquisition of metabolomic data.
Data analysis	Sequencing data was analyzed using custom R (3.6.1) and Python (3.6.15) scripts, using these tools: cutadapt (v2.3), FASTX-Toolkit (v.0.0.13), umitools (v0.3.3), bowtie2 (v2.3.5), salmon (v0.14.1), DEseq2 (1.24), STAR (2.7.1a), CellRanger (v3.0), and APAlog (https://github.com/goodarzilab/APAlog). El-MAVEN (0.7.0) and ProteoWizard (3.0.20315) were used for analysis of metabolomic data. RS-FISH (2.3.1) was used for quantification of smFISH data. Living Image (v4.7.3) was used to acquire in vivo imaging data with IVIS instrument (Perkin Elmer).

For manuscripts utilizing custom algorithms or software that are central to the research but not yet described in published literature, software must be made available to editors and reviewers. We strongly encourage code deposition in a community repository (e.g. GitHub). See the Nature Portfolio guidelines for submitting code & software for further information.

Data

Policy information about availability of data

All manuscripts must include a data availability statement. This statement should provide the following information, where applicable:

- Accession codes, unique identifiers, or web links for publicly available datasets
- A description of any restrictions on data availability
- For clinical datasets or third party data, please ensure that the statement adheres to our policy

All sequencing data produced in this study have been deposited in the GEO database under accession number GSE186641. Breast cancer data from the TCGA

research network analyzed in this study is available at: https://portal.gdc.cancer.gov/projects/TCGA-BRCA. Breast cancer data from the METABRIC dataset analyzed in this study was obtained from cBioPortal (https://www.cbioportal.org). CLIP-seq data from CLIPdb19 is available at: http://clipdb.ncrnalab.org. ENCODE datasets are available at: https://www.encodeproject.org. Hg38 (https://www.ncbi.nlm.nih.gov/assembly/GCF_000001405.40) has been used as the human genome reference sequence. Previously published datasets analyzed in this study are available under the following GEO accession numbers: GSE496493, GSE636054, GSE764887, GSE7763420, GSE3580021, GSE4582723, GSE5601024, GSE18664727, GSE6609228. Source data for Figs. 1d, 1f-h, 2f-i, 3b-e, 4d-f, 5a-c, 6a-e, 7b, 7d-e, and Extended Data Figs. 1h-l, 5a-d, 6a-m, 6pm have been provided as source data files.

Human research participants

Policy information about studies involving human research participants and Sex and Gender in Research.

Reporting on sex and gender	N/A
Population characteristics	N/A
Recruitment	N/A
Ethics oversight	N/A

Note that full information on the approval of the study protocol must also be provided in the manuscript.

Field-specific reporting

Please select the one below that is the best fit for your research. If you are not sure, read the appropriate sections before making your selection.

☒ Life sciences ☐ Behavioural & social sciences ☐ Ecological, evolutionary & environmental sciences

For a reference copy of the document with all sections, see nature.com/documents/nr-reporting-summary-flat.pdf

Life sciences study design

All studies must disclose on these points even when the disclosure is negative.

Sample size	Based on our previous work (Goodarzi et al., Nature, 2014; Goodarzi et al., Cell, 2015; Goodarzi et al., Cell, 2016), for in vivo experiments, mice were distributed into cohorts with 4-5 mice per cohort, which in NSG background is enough to observe a >2-fold difference with 90% confidence. For example, in MDA-LM2 cells in the same study, at t=33 days, average normalized signal was recorded as 291 and s.d. of 104, which suggests a cohort size of n=4.<br>For other experiments, no statistical methods were used to calculate sample size.
Data exclusions	No data were excluded from analysis in this study.
Replication	RT-qPCR was performed in a minimum of 3 biological replicates. Dose-response experiments were performed in 4 biological replicates. Other cell culture experiments were performed in a minimum of 3 biological replicates. Sequencing-based experiments were performed in 2 biological replicates.
Randomization	Mice for in vivo experiments were randomly assigned into cohorts. For other experiments (molecular biology), no randomization was performed.
Blinding	Investigators were blinded to group allocation during data collection and analysis.

Reporting for specific materials, systems and methods

We require information from authors about some types of materials, experimental systems and methods used in many studies. Here, indicate whether each material, system or method listed is relevant to your study. If you are not sure if a list item applies to your research, read the appropriate section before selecting a response.

Materials & experimental systems

n/a	Involved in the study
☐	☒ Antibodies
☐	☒ Eukaryotic cell lines
☒	☐ Palaeontology and archaeology
☐	☒ Animals and other organisms
☒	☐ Clinical data
☒	☐ Dual use research of concern

Methods

n/a	Involved in the study
☒	☐ ChIP-seq
☒	☐ Flow cytometry
☒	☐ MRI-based neuroimaging

Antibodies

Antibodies used	Rabbit anti-NQO1: Proteintech 11451-1-AP, Lot 2, 1:200 dilution<br>Biotinylated goat anti-rabbit IgG: Vector Labs BA-1000, 1:200 dilution<br>Mouse anti-HNRNPC: Santa Cruz Biotech sc-32308, Lot L1216, 5µg per confluent 15cm plate of cells<br>Rabbit anti-CTCF: Abcam, ab188408, Lot GR239192-3, 2µg antibody per 25µg chromatin
Validation	Rabbit anti-NQO1: Proteintech 11451-1-AP. This antibody has been validated in K562, HepG2, MCF-7, and human pancreatic cancer tissue per manufacturer's website (https://www.ptglab.com/products/NQO1-Antibody-11451-1-AP.htm).<br><br>Mouse anti-HNRNPC: Santa Cruz Biotech sc-32308. This antibody has been validated in Jurkat, MCF7, Hep G2, Sol8, NIH/3T3 and RPE-J whole cell lysates, and human kidney, ovary, and gall bladder tissue per the manufacturer's website (https://www.scbt.com/p/hnrnp-c1-c2-antibody-4f4).<br><br>Rabbit anti-CTCF: Abcam, ab188408. This antibody has been validated in HeLa, LLC, RAW 264.7, HEK-293, Hep G2, MCF7, C6, PC-12 and NIH/3T3 cells, as well as in human fetal brain, heart, kidney, and spleen lysate per the manufacturer's website (https://www.abcam.com/ctcf-antibody-epr18253-chip-grade-ab188408.html).

Eukaryotic cell lines

Policy information about cell lines and Sex and Gender in Research

Cell line source(s)	Human cell lines used in this study are available from ATCC:<br>MDA-MB-231 (ATCC HTB-26)<br>HEK293T (ATCC CRL-3216)<br>HCC1806 (ATCC CRL-2335)<br>BT-20 (ATCC HTB-19)<br>MDA-LM2 cell line, the lung metastatic derivative of MDA-MB-231, has been described (ref. 1 in the manuscript), and was a gift from Dr. Joan Massague.<br>HCC1806-LM2c cell line, the lung metastatic derivative of HCC1806, was a gift from Dr. Sohail Tavazoie.
Authentication	All cells were authenticated using short tandem repeat (STR) analysis following receipt (either from ATCC, or as a gift).
Mycoplasma contamination	All cells tested negative for mycoplasma contamination by PCR.
Commonly misidentified lines<br>(See ICLAC register)	No commonly misidentified cell lines were used.

Animals and other research organisms

Policy information about studies involving animals; ARRIVE guidelines recommended for reporting animal research, and Sex and Gender in Research

Laboratory animals	For all experiments involving animals, seven- to twelve-week-old age-matched female NOD/SCID gamma mice (Jackson Labs) were used. Mice were fed PicoLab® Rodent Diet 20 (#3005740-220) and housed in ventilated microisolator cages maintained at 68-74 degrees and 30-70% humidity, with no more than 5 animals per cage.
Wild animals	The study did not involve wild animals.
Reporting on sex	Animals used in this study were exclusively female, as breast cancer is a disease that predominantly affects females.
Field-collected samples	The study did not involve samples collected from the field.
Ethics oversight	The study protocol was approved by the University of California San Francisco IACUC committee (protocol AN179718-03F). For primary tumor experiments, the maximal tumor size permitted by UCSF IACUC of 20mm in any direction was not exceeded in this study.

Note that full information on the approval of the study protocol must also be provided in the manuscript.
